# Porous Ultralow‐*κ* sp^2^‐Bonded Boron Nitride Thin Films as Copper Diffusion Barriers

**DOI:** 10.1002/advs.76923

**Published:** 2026-07-30

**Authors:** Caiyun Liu, Ze Long, Zhongyuan Han, Yingying Guo, Yuning Ding, Tianhao Guo, Yuting Tang, Runzhi An, Haoran Ma, Jishan Liu, Hongwei Liang, Hong Yin

**Affiliations:** ^1^ State Key Laboratory of High Pressure and Superhard Materials College of Physics Jilin University Changchun People's Republic of China; ^2^ School of Integrated Circuits Dalian University of Technology Dalian People's Republic of China; ^3^ Shanghai Synchrotron Radiation Facility Shanghai Advanced Research Institute Chinese Academy of Sciences Shanghai People's Republic of China

**Keywords:** diffusion barrier capability, mechanically stable, porous BN, ultralow dielectric constant

## Abstract

Dielectric materials with low dielectric constant (*κ* < 2) are highly needed to overcome a series of problems caused by the decreasing size of microelectronics. An effective way to reduce dielectric constant is introducing void structure in dielectric materials, however challenging in mechanical strength and wetting properties. Herein, porous sp^2^‐bonded boron nitride (BN) films are directly grown on Si substrates via radio frequency magnetron sputtering. The Guinier plot analysis of grazing incidence small‐angle X‐ray scattering spectra and electron microscopy confirms the pore size of ∼1.85 nm. The porous BN (p‐BN) films show a band gap of ∼5.88 eV and a hydrophobic surface, ensuring the stability of dielectric properties. P‐BN films exhibit an ultralow‐*κ* of 1.77 at 100 kHz. These porous ultralow‐*κ* BN films are electrically robust and mechanically stable superior to the previously reported porous dielectrics and close to the referent dense amorphous BN and hexagonal BN. Additionally, the p‐BN films demonstrate notable diffusion barrier capability, effectively blocking Cu diffusion even with a thickness as low as 3 nm. This work provides a viable approach towards the controlled synthesis of porous ultralow‐*κ* BN films as versatile dielectrics for transistor gate barriers, passivation layers, and capacitor spacers in high‐performance electronics.

## Graphical abstract

Porous sp^2^‐bonded boron nitride (p‐BN) thin films with an ultralow dielectric constant (*κ* = 1.77) are directly grown on Si substrates via RF magnetron sputtering. The films feature uniform nanopores (1.85 nm), high mechanical stability, hydrophobicity, and excellent Cu diffusion barrier performance even at 3 nm thickness, offering a promising solution for advanced interconnect technologies.

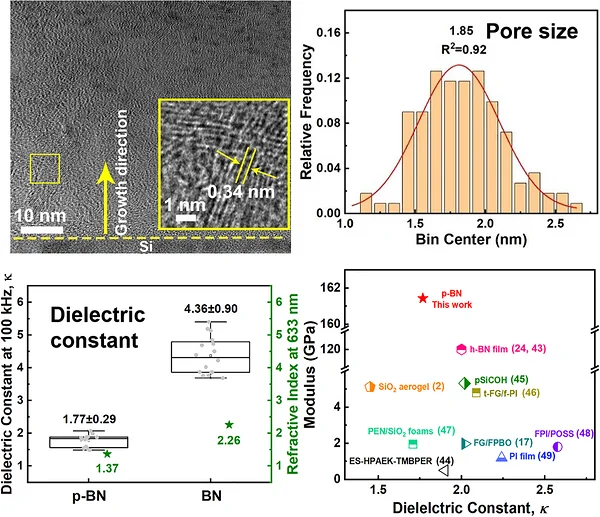

## 1 Introduction

With the rapid development of modern high‐performance multifunctional microelectronic integrated circuits (ICs), the resistance and capacitance (RC) delay that arises from the signal propagation through metal interconnections has become a fundamental obstacle to further enhancing the transistor speed, particularly associated with the increased packing density and the scaling down of device dimensions [1, 2]. State‐of‐the‐art strategies for reducing resistance and capacitance have involved exploiting copper (Cu) with lower resistivity to replace aluminum (Al) as metal interconnects [3, 4, 5] as well as electrically insulating materials with low dielectric constant (*κ*) for replacing the traditional dielectrics such as SiO_2_, HfO_2_, TiO_2_, and Al_2_O_3_ [6, 7]. Such low‐*κ* dielectrics can serve as transistor gates, capacitors, memory devices, and diffusion barriers, which are imperative, yet extremely challenging due to their high requirement for excellent electrical, mechanical, and thermal stability.

According to the recommendation from the International Roadmap for Devices and Systems (IRDS), ultralow‐*κ* dielectrics (*κ* < 2) are urgently needed concerning the interconnect development by 2028 [8]. Despite various attempts, most of the previously reported pore‐free materials show *κ* values higher than 2.5 [9, 10, 11, 12]. On the other hand, the introduction of porosity is considered one of the effective methods to achieve a lower dielectric constant [1, 2], where the interface polarization with vacuum or air is the determinant of the dielectric properties. However, highly porous dielectrics often suffer from integration challenges due to the degradation of mechanical strength and wetting properties. For instance, the film network connectivity could be disrupted due to the presence of mesopores or involving higher levels of packing voids [1, 13], which is detrimental to mechanical strength. This will cause film delamination following the mechanically demanding processes, such as chemical mechanical polishing, wire bonding, chip cutting, etc. [14, 15], eventually leading to device failure. Moreover, some porous materials exhibit high hydrophilicity because of their inherent hydrophilic nature, nanoporous inner structure, and the presence of hydrophilic moieties, which increases their dielectric constant and leakage current [16, 17]. In addition, their adhesion to substrates should not be ignored for further semiconductor manufacturing. Nevertheless, it is essential to develop alternative porous low‐*κ* materials that are compatible with complementary metal oxide semiconductor (CMOS) technologies, while also achieving substantial cost reductions, scalable production, and performance stability.

Boron nitride (BN) has drawn considerable attention for its potential applications as low‐*κ* dielectrics, that are benefited from its exceptional properties such as ultra‐wide band gap of ∼6 eV [18], high thermal conductivity [19], extraordinary chemical and thermal stability [20], high optical transparency in wide spectral range [21], and high dielectric breakdown strength of 7.8 MV/cm [22]. The dielectric constant of crystalline hexagonal BN (h‐BN) is in the range of 2–5 [23, 24, 25] recent reports of amorphous BN (a‐BN) have demonstrated promising lower *κ* values and excellent barrier capability for Co diffusion [26]. So far, low‐*κ* BN dielectrics have been widely synthesized in the form of ultrathin BN films over appreciable lateral dimensions [22, 24, 26, 27]. The involvement of porosity within BN films, which is expected to further reduce the dielectric constant, has yet to be relatively unexplored. In contrast to porous BN (p‐BN) powders already enabled many fascinating applications [28, 29, 30, 31, 32] the direct growth of p‐BN films over large areas on desired realistic substrates offers alternative, straightforward opportunities for technologically important practical applications, such as ultralow *κ* dielectrics. In addition, there remains a need for evaluating their crucial properties for practical applications as metal barriers.

Herein, we demonstrate the direct and controllable growth of porous ultralow‐*κ* BN thin films on silicon substrates using radio frequency (RF) magnetron sputtering method for dielectric applications, showing electrical, mechanical, and thermal robustness. The evolution of microstructure and phase of BN films with pulsed negative bias voltage reveals a clear transition from sp^2^ to sp^3^ hybridization, with the pore size significantly reduced. The optimal BN films with a pore size of ∼1.85 nm exhibit an ultralow dielectric constant of 1.77 at an operating frequency of 100 kHz and keep relatively constant at higher frequencies. These p‐BN films exhibit a band gap of 5.88 eV and a hydrophobic surface, also showing excellent mechanical stability compared with other porous materials. The properties of 3 nm thick p‐BN films are evaluated, showing an excellent capability of against Cu diffusion, and strong breakdown even at a higher temperature of 250°C. These results demonstrate the feasibility of depositing porous ultralow‐*κ* BN films directly on the desired substrate of interest, thus facilitating simple integration into the silicon industry at moderate cost and attainable film deposition approaches.

## 2 Results and Discussion

### 2.1 Synthesis and Characterization of Porous BN Thin Films

The p‐BN films have been prepared using RF magnetron sputtering method with a pulsed negative bias voltage. The growth detail is described in experimental section. A pore‐like structure is clearly observed in the films with the pore size significantly reduced in response to the enhanced pulsed negative bias accordingly (Figures S1–S3). Such morphological change presumably originates from the progressive enhancement in energetic bombardment of growth species induced by the negative bias. Considering that a large pore size is incompatible with advanced interconnects where the distance between adjacent metal wires may fall below 20 nm in the first metallization layers, we obtain p‐BN films with small pore sizes by adjusting the pulsed negative bias voltage to 100 V.

Figure 1a shows the surface morphology and microstructure of the p‐BN film, revealing film uniformity on a large‐area scale and good adhesion to the silicon substrates. The typical cross‐section high‐resolution transmission electron microscopy (HRTEM) image of the porous films clearly shows a crystal lattice with an average interplanar distance of ∼0.34 nm, corresponding to sp^2^‐BN (0002) (Figure 1b) [33]. Figure 1c displays the cross‐sectional selected area electron diffraction (SAED) of the p‐BN films. The complete uniformity of ring‐like h‐BN (0002) diffraction patterns suggests a random orientation, indicating that the films are polycrystalline structure. The B and N K edge electron energy loss spectra (EELS) shown in Figure 1d reveal the stronger π* peak and σ* energy‐loss features, confirming the existence of sp^2^ hybridization state of the typical h‐BN [34]. Figure 1e shows the Fourier transform infrared spectroscopy (FTIR) spectrum of the p‐BN film, in which two predominating absorbance bands at around 781 and 1381 cm^−1^ are associated with the out‐of‐plane B─N─B bending vibration and in‐plane B─N stretching vibration of typical sp^2^‐BN [35]. In addition, Raman spectroscopy of the porous films in Figure 1f reveals a sharp prominent peak located near 1369 cm^−1^, corresponding to the high‐energy phonon E_2g_ mode of sp^2^ B─N bond stretching vibration, especially for h‐BN [36]. Notably, the peak at 1450 cm^−1^ is attributed to the Si third TO mode from the substrate [37]. X‐ray photoelectron spectroscopy (XPS) further confirms that the B_1s_ and N_1s_ locating at 190.7 eV and 398.4 eV correspond to the sp^2^‐bonded B‐N bonds in the p‐BN film with a B/N ratio of 1.12 (Figure S4) [38]. It is worth mentioning that further enhancing the bias voltage leads to the formation of firm and dense BN films (Figure S4), eventually inducing a clearly phase transition from sp^2^ to sp^3^ hybridization at ∼170 V (Figure S5). In addition, X‐ray Reflectivity (XRR) was utilized to characterize the density of the porous and dense films, and the results are displayed in Figures 1g,h. By fitting the reflectivity curve, the density of the films can be obtained. The porous films exhibit a significantly smaller density of 1.41 g/cm^3^, as compared with that of dense BN films (2.28 g/cm^3^), which strongly implies the distinct difference in microstructures (Table S1).

**FIGURE 1:**
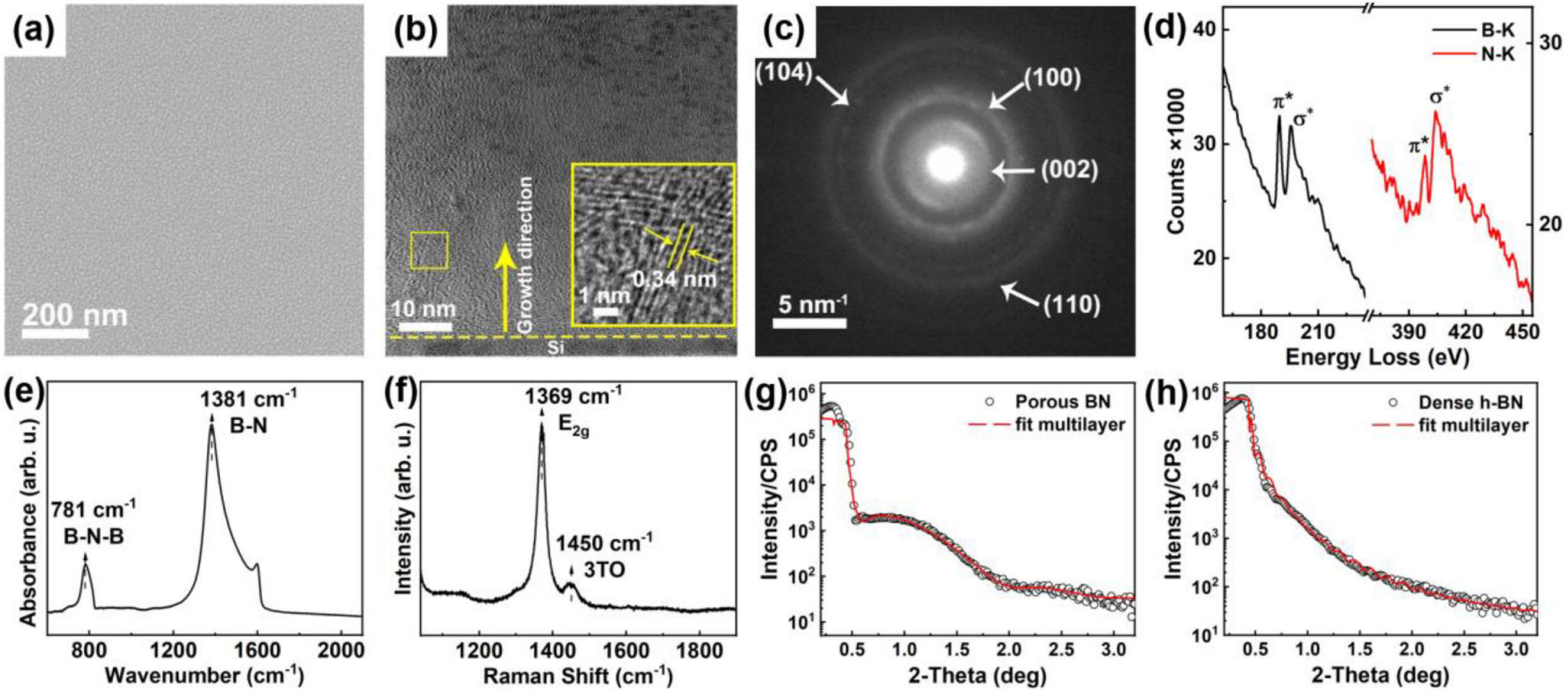
Crystal structural characterization of p‐BN films. (a) SEM image of the p‐BN films showing the surface morphology and microstructure. (b) Cross‐sectional HRTEM image of the p‐BN films, and the inset shows the lattice fringes. (c) SAED pattern with uniform ring‐like diffraction. (d) EELS spectra of B and N K‐edges. (e) FTIR spectrum with absorption bands at 781 and 1381 cm^−1^. (f) Raman spectrum showing the E_2g_ mode peak at 1369 cm^−1^. (g) and h) show the XRR profiles and the fitting curves of p‐BN and dense h‐BN films.

It is worth mentioning that by optimizing the substrate tilt angle and rotation during deposition, excellent uniformity and conformality of the p‐BN films were achieved on patterned Si substrates featuring trenches of different depths (Figures S6–S10 and Table S2).

### 2.2 Microscopic and Spectral Characterizations of Pores in BN

Generally, in the focused ion beam (FIB) process for cross‐sectional sample preparation, a platinum (Pt) layer is required to be plated on the surface of the BN films as a protective layer, and Pt constantly drops during the subsequent cutting and thinning process, so the pores in the film are inevitably filled with Pt (Figure S11). By observing the lattice of Pt (111) distributed in the holes, the pores can be indirectly visualized in the high‐resolution image, as shown in Figure 2a. From the higher‐resolution image, the sp^2^‐BN (0002) lattice is distributed around the holes (Figure 2b). Thus, an average pore size of ∼1.85 nm is obtained by statistical analysis of HRTEM (Figure 2c and Figure S12). Furthermore, the grazing‐small‐angle X‐ray scattering (GISAXS) measurement is conducted to shed light on the porous structures. A representative two‐dimensional (2D) GISAXS pattern is presented in Figure 2d, from which a one‐dimensional (1D) in‐plane profile is extracted at *α_f_* = 0.17°, where *α_f_* is the angle between the scattered beam and the film surface (Figure 2e). The scattering intensity decreases steeply. For mono‐scattered particle systems, the scattering intensity follows Guinier's law [2, 3],

(1) $$I\left(q\right)=I\left(0\right)exp\left(\frac{-R_{g}q^{2}}{3}\right)$$

where *I*(0) is the scattering intensity when the *q* = 0, *R_g_* is the average radius of gyration. For fitting the Guinier curve (Figure 2f) where a linear portion is obtained at a very small *q* value (the inset of Figure 2f), the average radius of gyration of the scattered particles in the sample is calculated to be approximately 0.71 nm. When the nanoparticles are spherical, the average radius of the nanopores, *R*, is expressed by

(2) $$R=\sqrt{\frac{5}{3}}\;R_{g}$$

**FIGURE 2:**
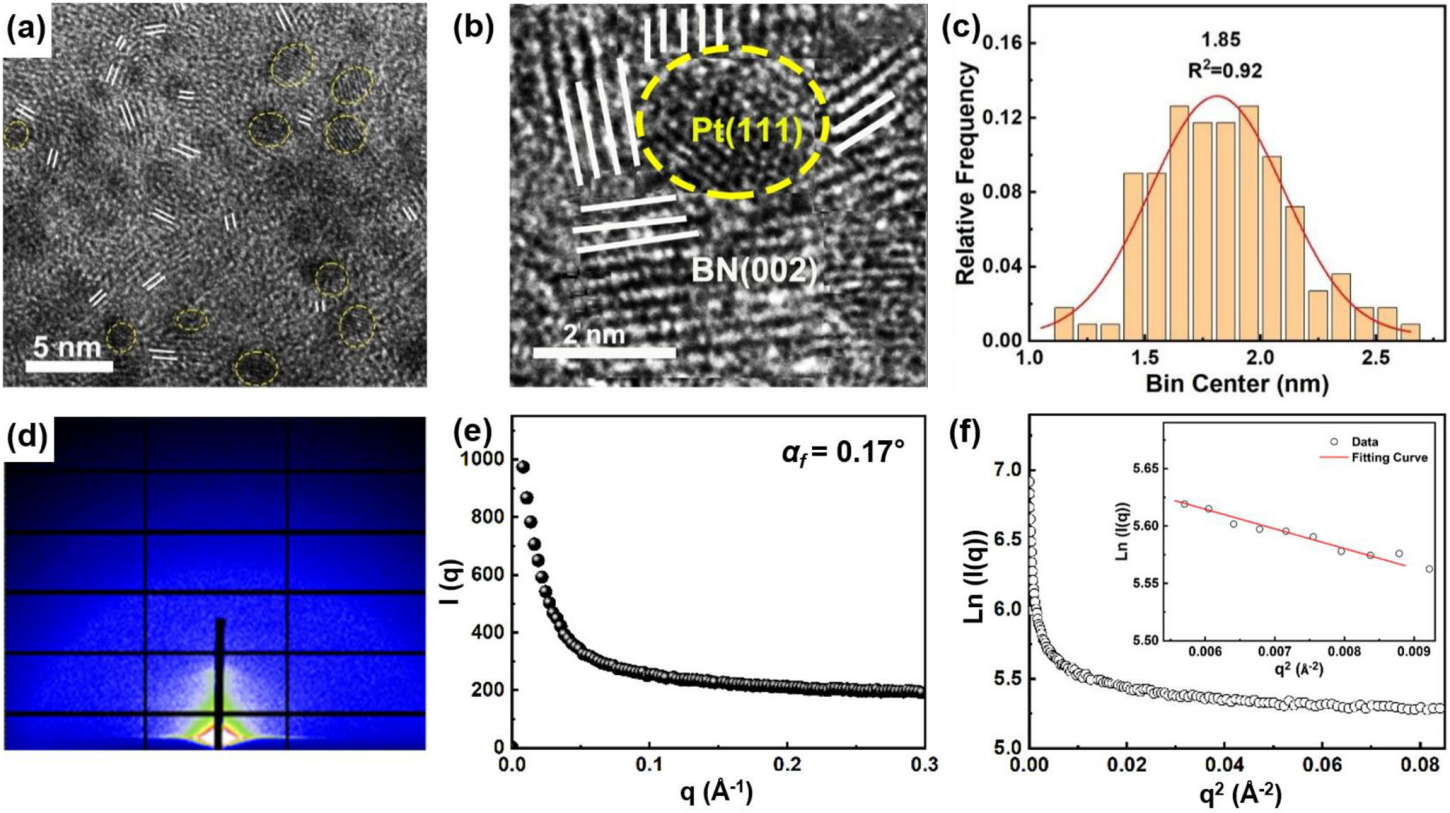
Pore structure characterization of the p‐BN films. (a) Cross‐sectional HRTEM image of the p‐BN film and (b) a higher magnification image. The yellow dashed line outlines the range of Pt particles. (c) Pore size distribution histogram obtained from HRTEM image analysis. (100). (d) Representative 2D GISAXS pattern of the p‐BN film and (e) an in‑plane scattering profile extracted at *α_f_*  =  0.17°. (f) Guinier curve and the fitting curve of the low‑*q* scattering data (inset).

The average radius of the nanopores (*R*) is 0.92 nm, and the average diameter is ∼1.84 nm, which agrees well with the HRTEM results.

### 2.3 Dielectric and Mechanical Performance of Porous BN Films

Furthermore, the dielectric properties of p‐BN films are investigated using a metal‐insulator‐metal (MIM) device. The BN films were grown on a heavily doped n‐type Si substrate with a sample size of approximately 1 × 1 cm^2^. Cu contacts (0.55 mm diameter) were magnetron sputtered on the film surface, and a bottom Cu electrode on the backside of Si substrate. The detailed Cu/BN/Si/Cu device configuration is illustrated as inset in Figure 3a. The capacitance–voltage (C–V) characteristics of BN is performed to evaluate the relative dielectric constant. The relative dielectric was calculated by $\kappa_{m}=\frac{dC_{m}}{A\kappa_{0}}$, where *d* is the thickness of the BN films, *C_m_* represents the measured capacitance, *A* is the area of the Cu electrode, *κ_m_* and *κ_0_* (8.854 × 10^−12^ F/m) are the relative dielectric constant of the matrices and the vacuum dielectric constant, respectively. Considering that n‐type Si is also capacitive and its resistivity is higher as compared to the commonly used metal electrodes, therefore the interference of capacitance value from Si substrate should be corrected using $\frac{1}{C_{m}}=\frac{1}{C_{f}}+\frac{1}{C_{s}}$ [39], where *C_f_* represents the capacitance of the BN film, and *C_s_* is contributed by the Si substrate. Meanwhile, the capacitance of Si substrate is measured using Cu/n‐Si/Cu configuration with the same metal electrodes.

**FIGURE 3:**
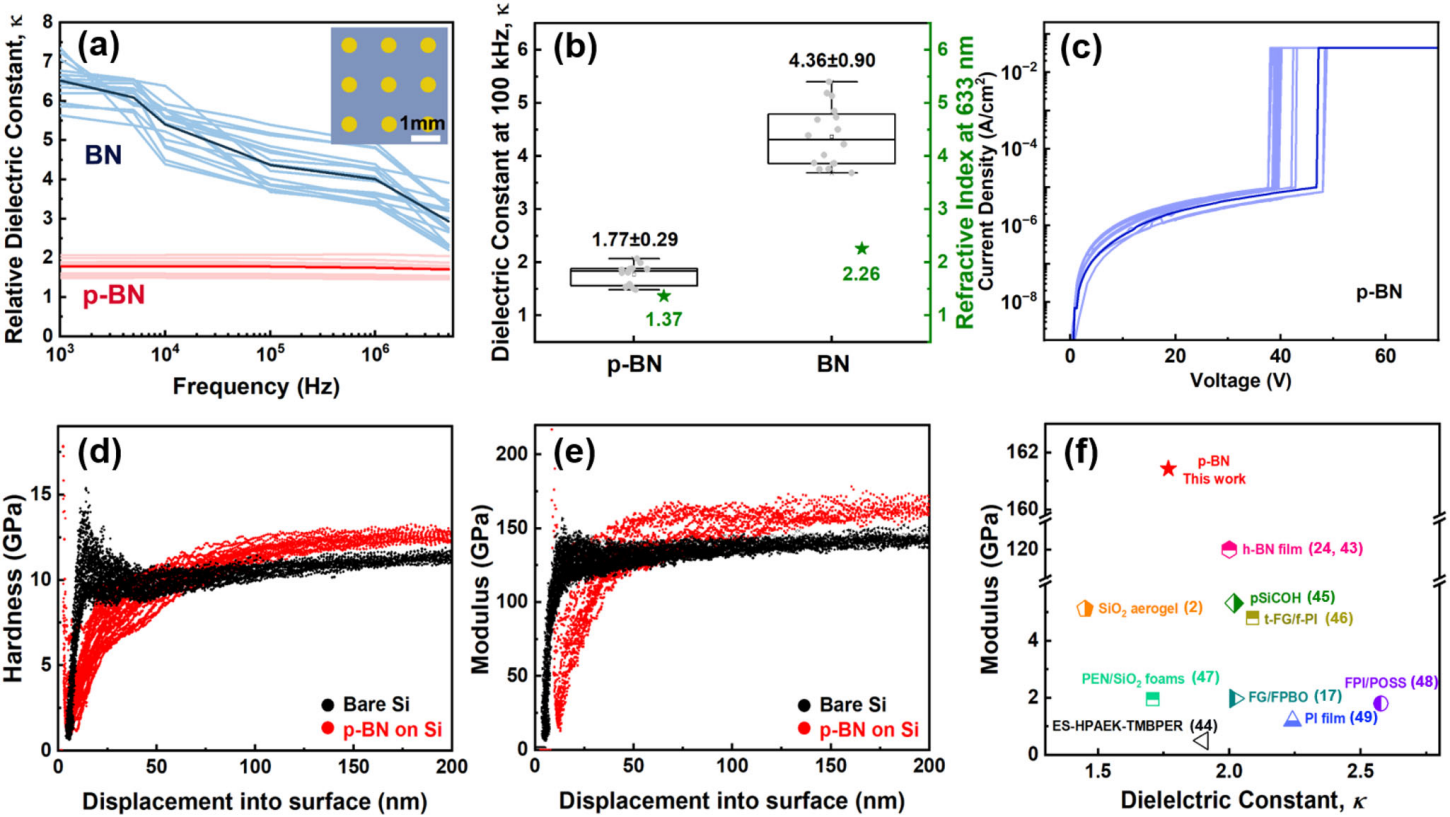
Electrical and mechanical properties. (a) Relative dielectric constant as a function of frequency after correction. Thick red and blue lines denote averages; the inset illustrates MIM device structure. (b) Statistical distribution of dielectric constants measured at 100 kHz for p‐BN and dense h‐BN. The box indicates a region with a 25% and 75% distribution relative to the average value, and the top and bottom bars mean maximum and minimum values. The number of devices measured for p‐BN is 12, for dense h‐BN is 15. The refractive index at 633 nm is shown by the green five‐pointed star symbol. (c) Summary of repeatedly measured breakdown characteristic curves for p‐BN films. (14) (d) and (e) Nanoindentation results showing that the deposition of p‐BN on Si substrates leads to an increase in surface hardness and modulus. The number of areas measured on one sample is 30 to get an average. (f) Scatter plot with *κ*‐value and modulus [2, 17, 24, 43, 44, 45, 46, 47, 48, 49].

The relative dielectric constants after correction for both porous sp^2^ BN and dense h‐BN films at different frequencies are shown in Figure 3a. It can be seen that the *κ* value for the porous film is stable in the frequency range of 1 kHz—5 MHz; however, the *κ* value for dense film dramatically decreasing with frequency, which corresponds to the strong dielectric relaxation [40]. The dielectric constant at different frequencies of the porous and dense BN films is listed in Table S3. It is significant that the dielectric constant of p‐BN (*κ* = 1.77 at 100 kHz) is remarkably lower than that of the dense h‐BN (*κ* = 4.55 at 100 kHz) under identical test conditions. The ultralow dielectric constant of the p‐BN films is cross‐checked by a PARSTAT 3000A electrochemical workstation in the wider frequency range, a similar *κ* value of ∼1.63 at 100 kHz is also obtained, and the dielectric loss factor is decreased to 0.001 at 7 MHz (Figure S13). The distribution of the measured values and the corresponding error bars at 100 kHz are summarized in Figure 3b. The ultralow dielectric constant of the p‐BN film is due to the presence of a pore structure, which reduces the number of polarized molecules per unit volume in the dielectric substance [41], rather than grain boundaries (Figure S14). The refractive index at a wavelength of 633 nm measured through spectroscopic ellipsometry is 1.37 and 2.26 for porous and dense BN film, implying the *κ* values of 1.88 and 5.10, respectively. Thus, the *κ* value is closely consistent with the low dielectric constant of the p‐BN films.

Moreover, the surface current–voltage (*I–V*) measurements suggest that the p‐BN films are electrically stable in response to the operating temperatures from room temperature up to 473 K, and the symmetry in *I–V* curves clearly implies the film uniformity for the porous sample (Figure S15a). Meanwhile, a vertical sandwich‐type device is constructed to measure the electrical breakdown strength of porous films, which is extracted by measuring the current density versus the applied bias. The results in Figure 3c show that the average breakdown voltage of the p‐BN film is 41 V, corresponding to a breakdown field strength of 4.1 MV/cm. The p‐BN exhibits an electrical breakdown strength of 4.1 MV/cm, lower than the dense h‐BN of 5.8 MV/cm (Figure S15d). Considering that the film thickness is about 100 nm, the p‐BN film and dense h‐BN exhibit exceptionally low leakage current density values of 1.17 µA/cm^2^ (p‑BN) and 1.15 µA/cm^2^ (h‑BN) at 10 V, respectively. Thus, these dielectric properties of p‐BN films demonstrate their potential as low‐*κ* dielectrics.

On the other hand, a porous structure is often accompanied with reduction of density, consequently spoiling the mechanical strength. We also measured the mechanical properties of the p‐BN films to confirm their strength, and the results of nanoindentation measurements shown in Figure 3d,e and Figure S16. Although the presence of pores slightly deteriorates the mechanical properties of dense BN, the hardness and modulus values of the porous films are estimated at 11.7 and 161.4 GPa, respectively, which are higher than those of bare Si (10.5 and 148.9 GPa). The mechanical behaviors of both porous and dense BN films remain similar in accordance to the loading, which are mainly affected by the Si substrate beneath. Overall, the dielectric constant and mechanical properties for p‐BN film obtained in this work are compared to the porous low‐*κ* materials reported in literature and the results are summarized in Table S4. Notably, by comparing the scatter plot of *κ*‐value and Young's modulus as shown in Figure 3f, it is found that the p‐BN films in our work exhibit ultralow dielectric constant and exceptionally high modulus, which is significantly higher than the previously reported porous low‐*κ* dielectrics and close to the continuous a‐BN and h‐BN. In addition, the water contact angle of ∼110.7° is estimated for the p‐BN films, significantly higher than that of the dense h‐BN film (∼88.9°) (Figures S17–S18). Such hydrophobicity prevents the adsorption of water vapor and other environmental substances, which would otherwise negatively impact the electrical performance and mechanical integrity of the device, thereby improving device stability (Figures S19–S21, Table S5) [17, 42]. More importantly, the dielectric properties, hydrophobicity, and mechanical stability of p‐BN films are improved as compared to the commercial SiO_2_ films (Figure S22).

### 2.4 Diffusion Barrier Properties of Porous BN Films

To further demonstrate the device applicability and performance, the diffusion barrier properties of the p‐BN films are evaluated. The presence of pores in BN films is tested for the blocking ability of a metallic Cu film with ∼100 nm thickness, which was deposited on the BN film surface forming a Cu/BN/Si structure. The cross‐sectional SEM images with the corresponding electron diffraction spectral (EDS) mapping (Figure S23) confirm that the metallic Cu has not diffused into the BN film through the pores at room temperature. Further annealing the Cu/p‐BN/Si devices in vacuum for 1 h at 600°C causes no observable diffusion; whereas severe diffusion of Cu into Si occurs when TiN is used as the barrier layer (Figure S24).

To evaluate the time‐dependent barrier properties, we further deposited 100 nm Cu on the p‐BN ultrathin films with the thickness of 3 nm and 5 nm, respectively. Figure 4a and the inset show cross‐sectional HRTEM images of the 3 and 5 nm thick p‐BN layers, while Figure 4b shows the FTIR spectra of p‐BN films. The characteristic peaks located at 781 and 1381 cm^−1^ confirm the structure of h‐BN, while the comparison of the FTIR spectral intensities indirectly verifies the film thickness. Figure 4c,d presents the secondary ion mass spectrometry (SIMS) depth profiles of the Cu/p‑BN/Si stack after vacuum annealing at 300°C for 30 min. The clearly separated distribution curves of Cu and Si demonstrate that even an ultrathin p‑BN layer of only 3 nm can effectively block the thermal diffusion of Cu.

**FIGURE 4:**
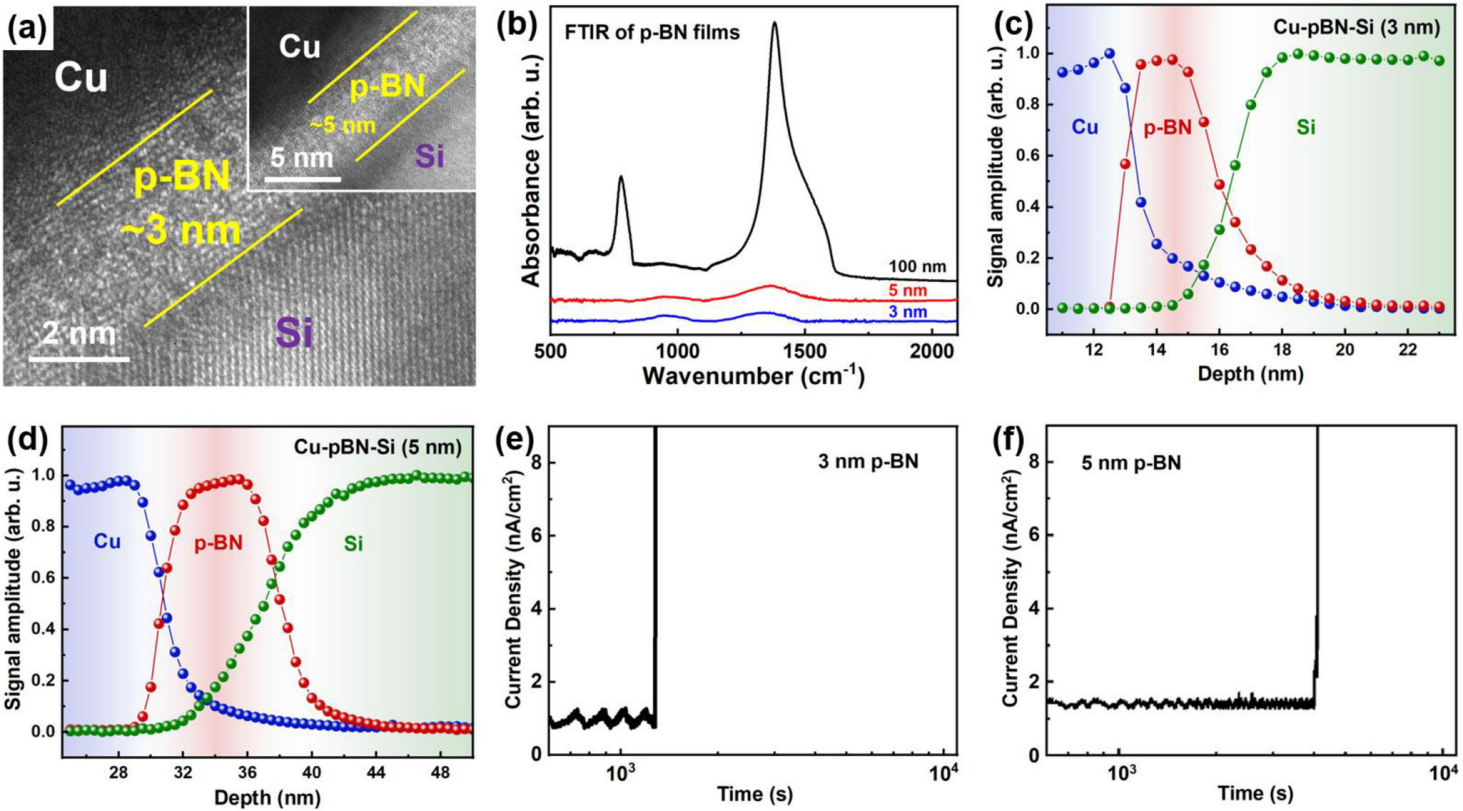
Characterization of the barrier performance of 3 and 5 nm p‐BN films. (a) Cross‐sectional HRTEM image of the Cu/p‐BN (3 nm)/Si stack. The inset shows the cross‐sectional image of the 5 nm p‐BN. (b) FTIR spectra of p‐BN films with different thicknesses. (c) and (d) SIMS depth profiles of the 3 and 5 nm Cu/p‐BN/Si stacks. Due to the barrier effect of p‐BN, no diffusion of Cu into the Si layer is observed. (e) and (f) Time‐dependent dielectric breakdown curves for devices with 3 and 5 nm thick p‐BN films, respectively.

Figure 4e,f shows the time‐dependent dielectric breakdown measurements based on the structure of Cu/p‐BN/Si/Cu under 4 MV/cm at 250°C, in which the current evolves with time for the devices fabricated using p‐BN films with different thickness of 3 nm and 5 nm. Under a positive bias applied, Cu ions pass through the p‐BN layer and the current increases rapidly. Due to the limitation for thickness, the time to failure of the 3 nm thick porous films is smaller than that of the 5 nm thick film. Thus, these results strongly suggest that the low‐*κ* p‐BN thin films can serve as the diffusion barrier.

## 3 Conclusion

In summary, the porous sp^2^‐bonded BN films with ultralow‐*κ* value of 1.77 at an operating frequency of 100 kHz were directly grown on the Si (100) substrates by RF magnetron sputtering. The average pore size of ∼1.85 nm can be regulated by pulsed negative bias voltages. These porous ultralow‐*κ* BN films are relatively electrically robust and mechanically stable, superior to the previously reported porous dielectrics and close to the continuous a‐BN and h‐BN. More importantly, the p‐BN films exhibit significant blocking diffusion capability even at a thickness at 3 nm. These results highlight the feasibility of depositing porous ultralow‐*κ* BN films directly on desired substrate by RF magnetron sputtering method, in terms of materials processing and industrial adaptability. Our work thus demonstrates the potential of these porous sp^2^‐BN as low‐*κ* dielectric layers for high‐performance electronics.

## 4 Experimental Section

### 4.1 Growth Procedure of Porous BN Films

The p‐BN films were grown on (100)‐oriented n‐type single‐crystalline silicon (12–30 Ω·cm, 300 µm thick) substrates using RF (13.56 MHz) magnetron sputtering method. High‐temperature sintered BN target (60 mm in diameter) was used. A RF power of 120 W was applied in all the cases. The target was positioned 8 cm away from substrate holder, which was electrically grounded. The target was sputter‐cleaned for 3 min prior to deposition and the substrate temperature was kept at 600°C. The background pressure was maintained below 3 × 10^−5^ Pa before introducing the working gases of Ar/N_2_ mixture with a flow rate of 50 sccm each. All the depositions were last for 3 h under 2 Pa working pressure. The negative bias voltage with a pulse frequency of 40 kHz was varied (0–100 V) to prepare the p‐BN films. The dense h‐BN film is deposited at 140 V pulsed negative bias voltage.

### 4.2 Material Characterization

Fourier transformed infrared spectroscopy (FTIR, Nicolet iS20) in absorbance mode with resolution of 4 cm^−1^ within the range of 400–4000 cm^−1^ and Raman spectra (Raman, Lab RAM HR Evolution Raman spectroscopy) with laser excitation wavelength of 473 nm were utilized to investigate chemical bonding structure and crystal quality of BN films. The surface morphology and the thickness of BN films were measured with scanning electron microscope (SEM, Magellan 400). The high‐resolution transmission electron microscopy (HRTEM, JEM‐2200FS and JEM‐F200) was studied to identify the porous structure of the films. Scanning transmission electron microscope (STEM, JEM‐ARM300F) was used to conduct EDS mapping. The X‐ray photoelectron spectroscopy (XPS, Thermo Scientific NEXSA) measurements were conducted to obtain the chemical structure. Contact angle measurement was carried on using a video optical contact angle meter (XGCAMC33) in a range of 0°–180° with measurement resolution of 0.01°. The optical characteristics of the p‐BN films were investigated by UV‐visible spectroscopy (UV‐vis, Thermo‐Fischer Evolution One plus). The wavelength ranges from 190 to 1100 nm with a resolution of 0.2 nm. The refractive index was investigated by spectroscopic ellipsometry (SE, UVISEL‐2) with XYZ automated 3D sample stage. Ellipsometry measurement were carried out in the wavelength range 200–800 nm with a resolution of 2 nm at incidence angles of 70°. The optical properties of both films were determined using the software of DeltaPsi 2 from testing to analysis. The density of the films was obtained by High‐Resolution Thin Film X‐Ray Diffractometer (HR TF‐XRD, Bruker D8 Discover). The measured X‐ray Reflectivity (XRR) data was fitted using the Bruker software Leptos to obtain the density, roughness and film thickness. Grazing‐small‐angle X‐ray scattering (GISAXS) measurements were carried out at the beamline BL16B1 of the Shanghai Synchrotron Radiation Facility (SSRF), China. A monochromatized X‐ray radiation source of λ = 0.124 nm (λ, wavelength) and a two‐dimensional charge‐coupled device (2D CCD) detector (Pilatus 2 M) were used. The sample‐to‐detector distance was 1910 mm. The incidence angle *α_i_* of each X‐ray beam was set at 0.2°. The hardness and modulus values of the porous BN film were investigated by Keysight NanoIndenter (G200). We conduct a Continuous Stiffness Measurement (CSM) dynamic measurement technique of the porous BN films. The surface approach velocity is 10 nm/s, strain rate target is 0.05 /s, frequency target is 45 Hz. Thirty points were randomly selected and the average values were compared, the data of Si substrate as reference. The elemental depth profiles of the Cu/p‐BN/Si, Cu/TiN/Si and Cu/Si devices were measured using secondary ion mass spectrometry (SIMS, PHI nanoTOF 3). A 30 keV Bi_3_^2+^ primary ion beam was used, and sputtering was performed synchronously over an analysis area of 0.04 mm^2^ during the measurement.

### 4.3 Electrical Characterization

I–V curves were measured from 300 to 473 K using a Keithley 2450 on the BN films grown on quartz substrates using four‐point method. Four 0.55 mm diameter Ti/Au top electrodes at the corner edge of the sample were used as metal contacts. The temperature dependent resistance measurement was performed using a Hall system (C‐Mag Vari‐9) from 10 to 300 K. The capacitance‐voltage (C‐V) characteristics of the films in metal/BN/Si/metal stacks were measured using Agilent B1505A Power Device Analyzer/Curve Tracer. The impedance spectra and capacitance‐frequency (C‐f) characteristics of the films in metal/BN/Si/metal stacks were measured using PARSTAT 3000A electrochemical workstation. A 100‐nm‐thick Cu electrode pattern each with 0.55 mm diameter was deposited over the BN/Si stack to form Cu/BN/Si/Cu device structure. At the same time, the same Cu electrodes were prepared on a bare n‐Si substrate to form Cu/Si/Cu stack as reference. After the device fabrication, capacitance‐voltage units in the Power Device Analyzer/Curve Tracer were used to perform the C‐f measurements. The C‐f measurements were carried on in the frequency range 1 kHz–5 MHz.

### 4.4 Statistical Analysis

Statistical analyses were performed using Origin software. All data points were included in the analyses without exclusion. For each experiment, values obtained from independent replicates were averaged prior to subsequent statistical analysis. Data are presented as mean ± standard deviation. Sample sizes for each analysis are reported in the corresponding figure legends either in text or indicated as (n).

## Author Contributions

**Yingying Guo**: investigation. **Caiyun Liu**: methodology, investigation, visualization, writing – original draft. **Yuting Tang**: investigation. **Ze Long**: methodology, investigation, visualization, writing – original draft, writing – review and editing. **Yuning Ding**: investigation. **Hong Yin**: conceptualization, methodology, supervision, writing – review and editing. **Tianhao Guo**: investigation. **Jishan Liu**: investigation. **Zhongyuan Han**: investigation. **Hongwei Liang**: conceptualization, methodology, supervision, writing – review and editing. **Haoran Ma**: investigation. **Runzhi An**: investigation.

## Funding

This research was supported by the National Natural Science Foundation of China (Grant Nos. 12335011, 12505340), Fundamental and Interdisciplinary Disciplines Breakthrough Plan of the Ministry of Education of China (JYB2025XDXM107), and Key R&D Project of Scientific Development Department of Jilin Province of China (20240302099GX).

## Conflicts of Interest

The authors declare no conflicts interest.

## Supporting information




**Supporting File**: advs76923‐sup‐0001‐SuppMat.docx.

## Data Availability

The data that support the findings of this study are available from the corresponding author upon reasonable request.
