# Supplementary material for: Porous Ultralow‐κ sp2‐Bonded Boron Nitride Thin Films as Copper Diffusion Barriers
Source: Adv Sci (Weinh). 2026 Jul 30:e76923. Online ahead of print. doi: 10.1002/advs.76923 (PMC13423504; doi:10.1002/advs.76923)
Supplement: Supplementary file 1 — Supporting File: advs76923‐sup‐0001‐SuppMat.docx. [file ADVS-9999-e76923-s001.docx]

Supporting Information

Porous ultralow-*κ* sp^2^-bonded boron nitride thin films as copper diffusion barriers

*Caiyun Liu, Ze Long, Zhongyuan Han, Yingying Guo, Yuning Ding, Tianhao Guo, Yuting Tang, Runzhi An, Haoran Ma, Jishan Liu, Hongwei Liang*, Hong Yin**

The Supporting Information includes:

Figure S1 to S24

Table S1 to S5

References 1 to 20

**1. Figures**


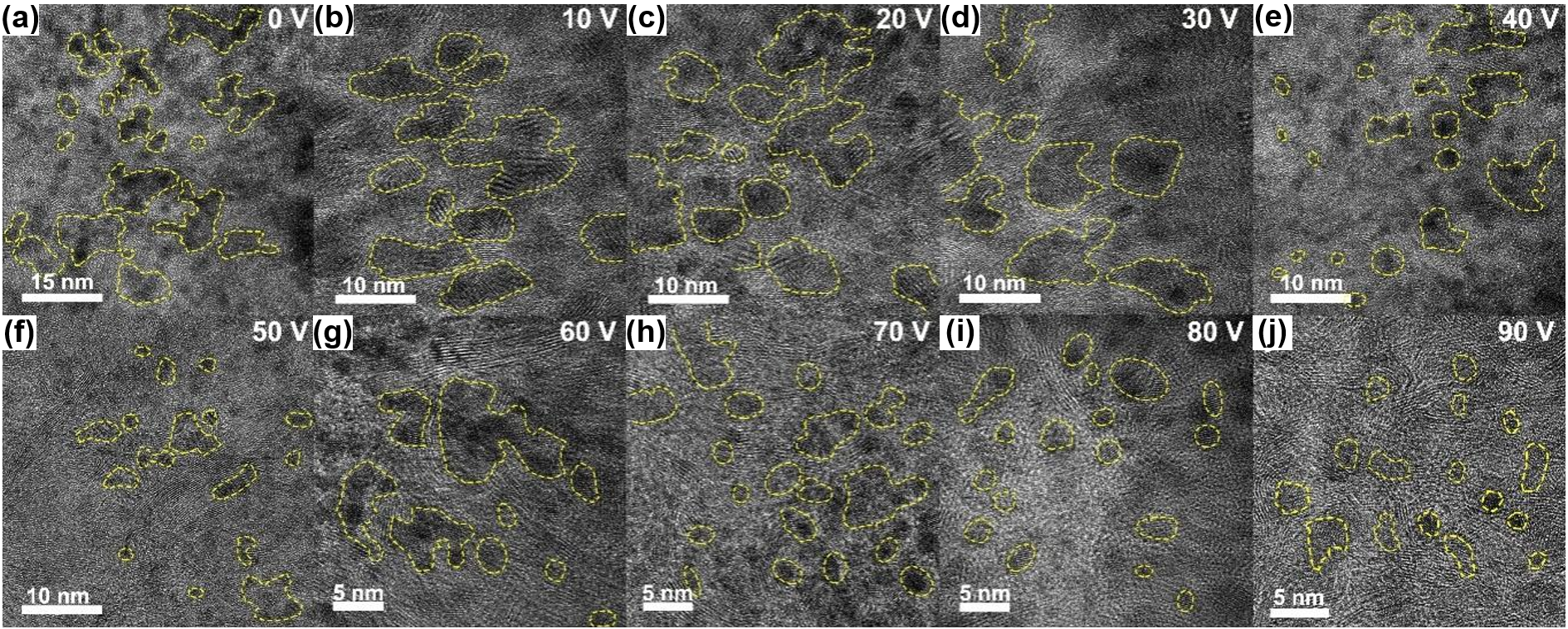


**Figure S1. HRTEM images of the porous BN (p-BN) films.**

a)-j) The cross-sectional HRTEM images of the p-BN films deposited with the pulsed negative bias voltage of 0-90 V.


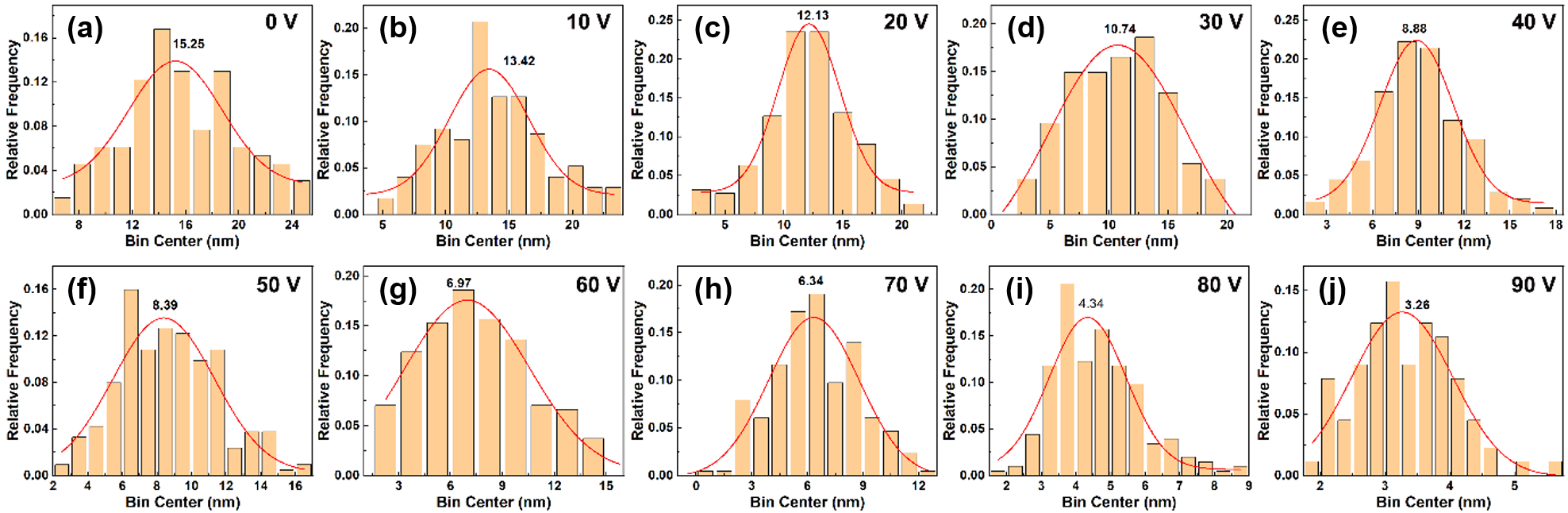


**Figure S2. Pore size statistics of the p-BN films**

a)-j) Pore size distributions of p-BN films prepared at different pulse negative bias voltages, statistically analyzed using HRTEM images. The pore size decreases from 15.25 nm to 3.26 nm as the pulsed negative bias increases from 0 to 90 V. (100)


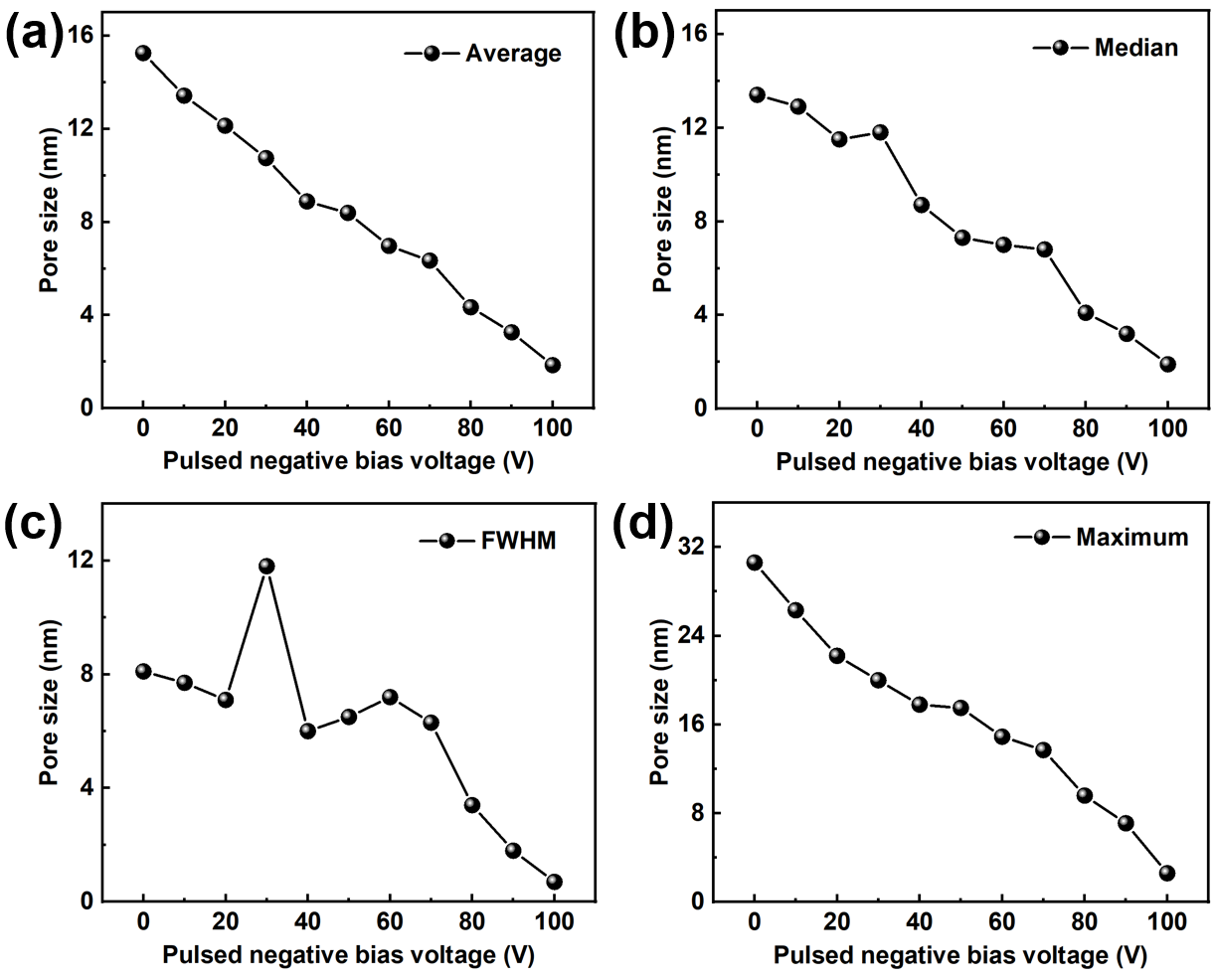


**Figure S3. Pore size statistics of the p-BN films**

Statistical plots showing the variation of a) average pore size, b) median pore size, c) FWHM of Gaussian distribution, and d) maximum pore size with bias voltage for the p-BN films. The pore size of p-BN decreases gradually with increasing negative bias voltage. (100)


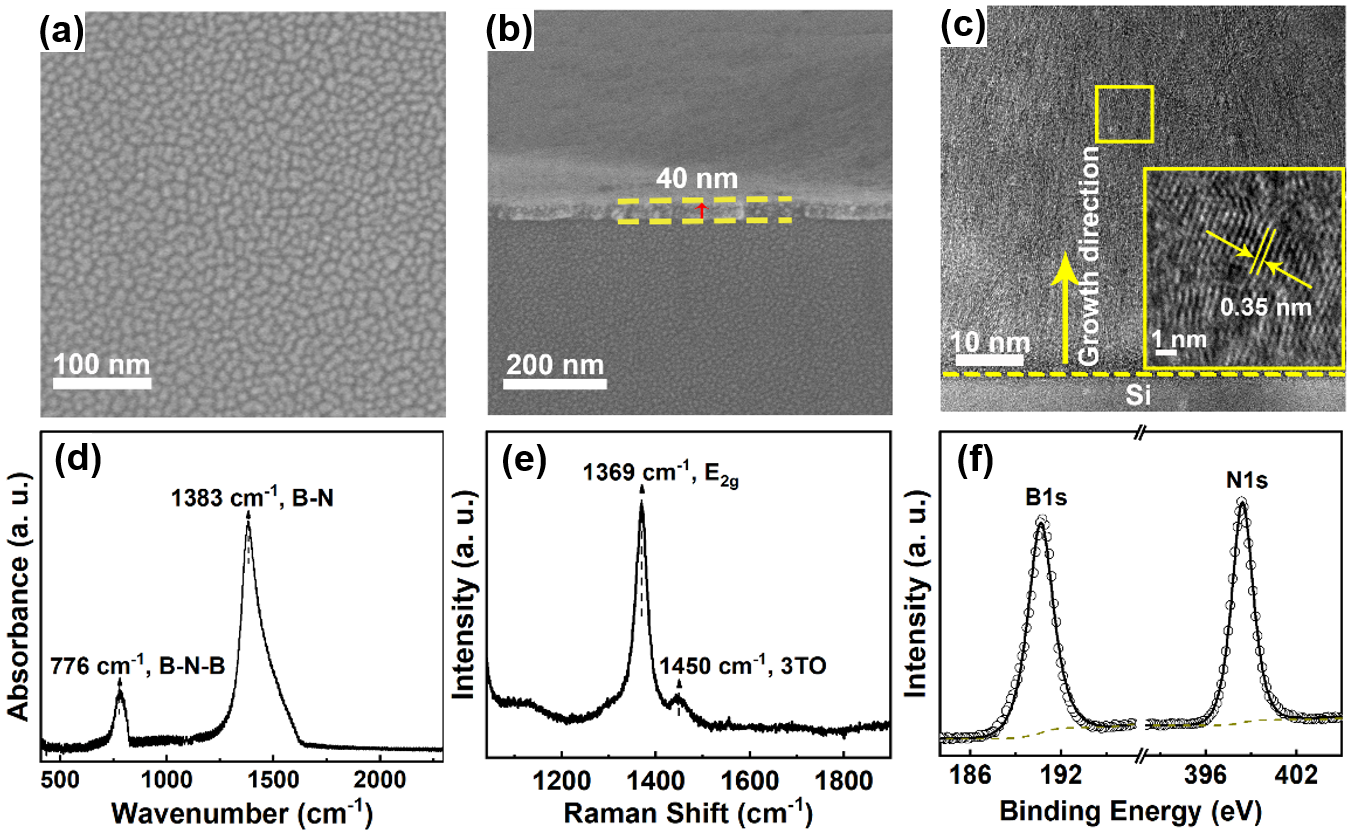


**Figure S4. Characterization of the dense h-BN film**

a)-b) Top and cross-sectional SEM images. c) HRTEM image. d) FTIR spectrum. e) Raman spectrum. f) B1s and N1s spectrum of the dense h-BN film deposited at 140 V.


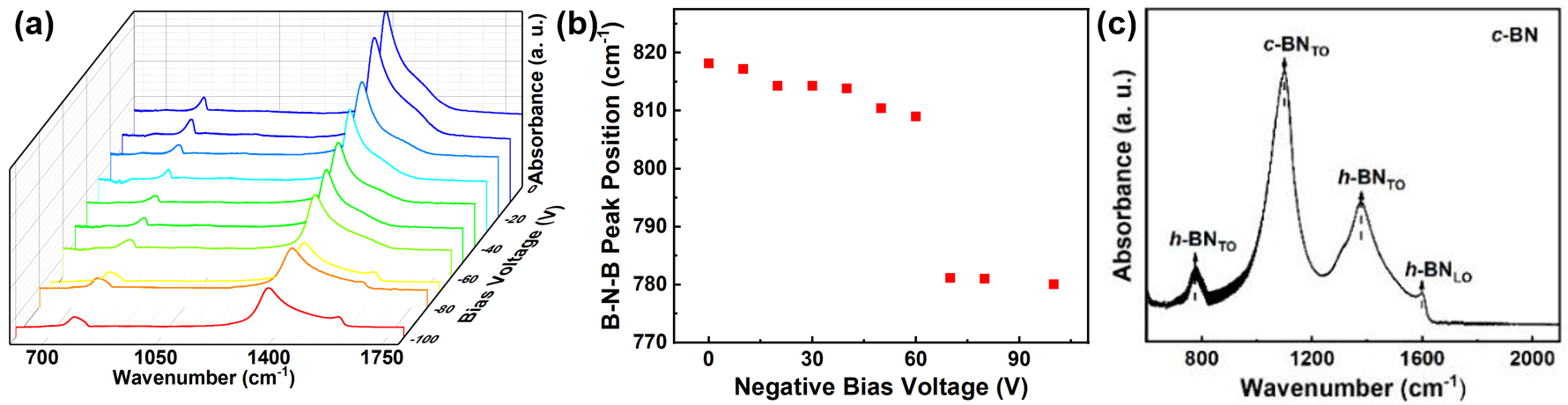


**Figure S5. FTIR spectra of the p-BN films deposited with different bias voltages.**

a) FTIR spectra of the p-BN films deposited with the pulsed negative bias voltage of 0-100 V. b) The B-N-B vibration peak position is changed from 819 cm^-1^ to 782 cm^-1^ due to increased stress with the negative bias voltage increased from 0 V to 100 V. c) FTIR spectrum of the p-BN grown at a bias voltage of 170 V, indicating a clear phase transition from sp^2^ to sp^3^ hybridization.

**
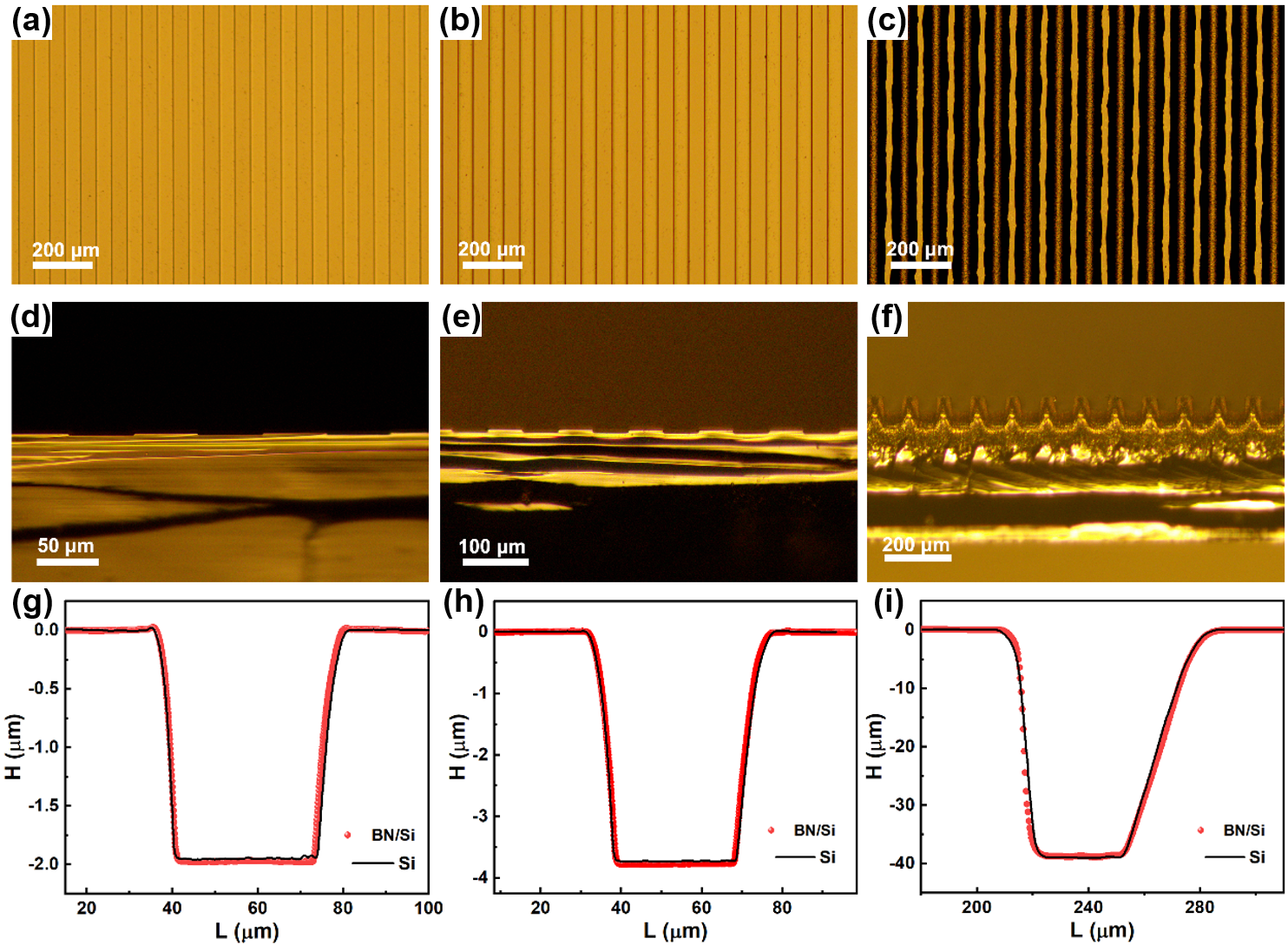
**

**Figure S6. The trench structures of Si substrate and p-BN films.**

a)-c) Surface and d)-f) cross sectional optical microscope images of trenched Si substrates with depths of 1.9 μm, 3.7 μm, and 37.7 μm. g)-i) Step profiler curves measured before and after deposition of p‑BN films on the trenched substrates, demonstrating the good coverage and conformality of the films grown by pulsed magnetron sputtering. The trench structures simulate realistic non‑planar features encountered in integrated circuits, and the results confirm the uniformity of p‑BN films over high-aspect-ratio topography.


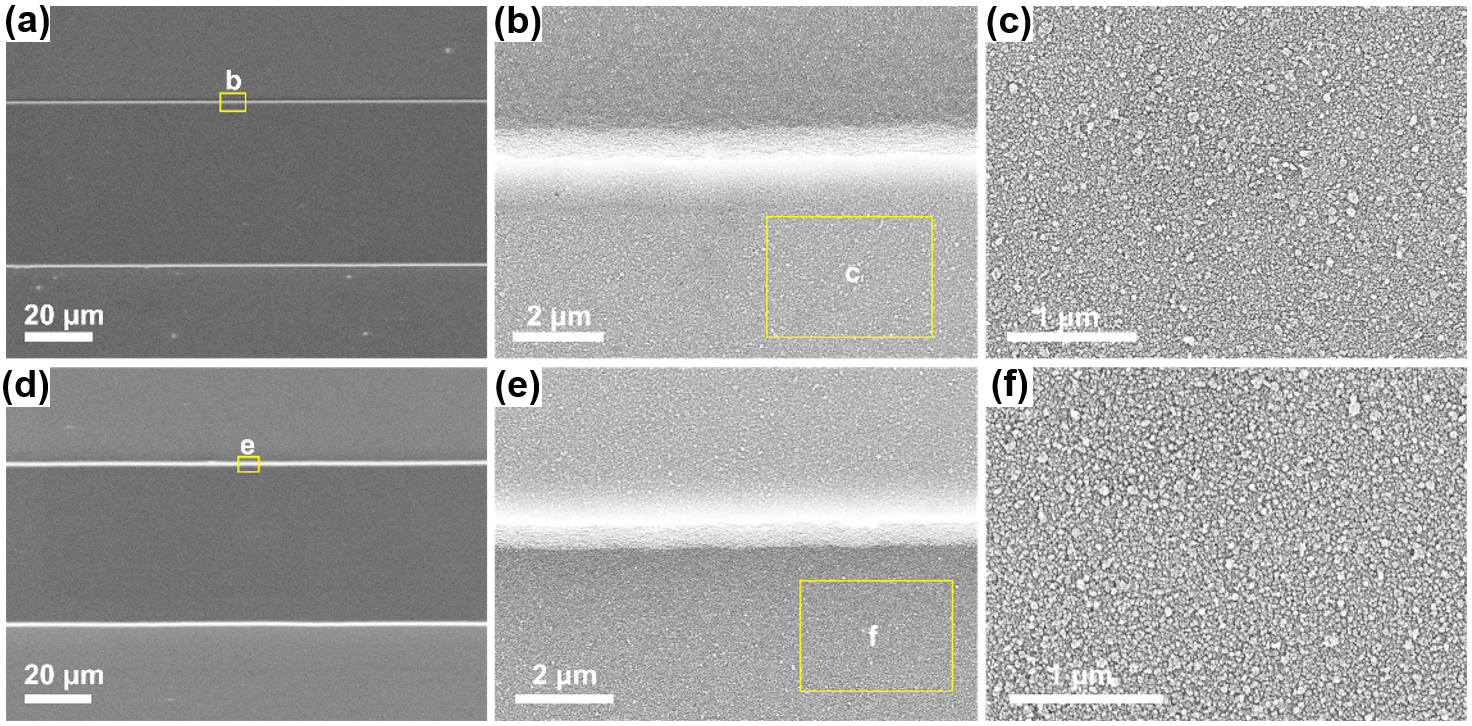


**Figure S7. Surface SEM images of the p-BN films (1.9 μm and 3.7 μm).**

Surface SEM images of the p-BN films deposited on the trenched Si substrate with the depth of a)-c) 1.9 μm, and d)-f) 3.7 μm, respectively. b) is marked area in a), c) is marked area in b), e) is marked area in d) and f) is marked area in e).


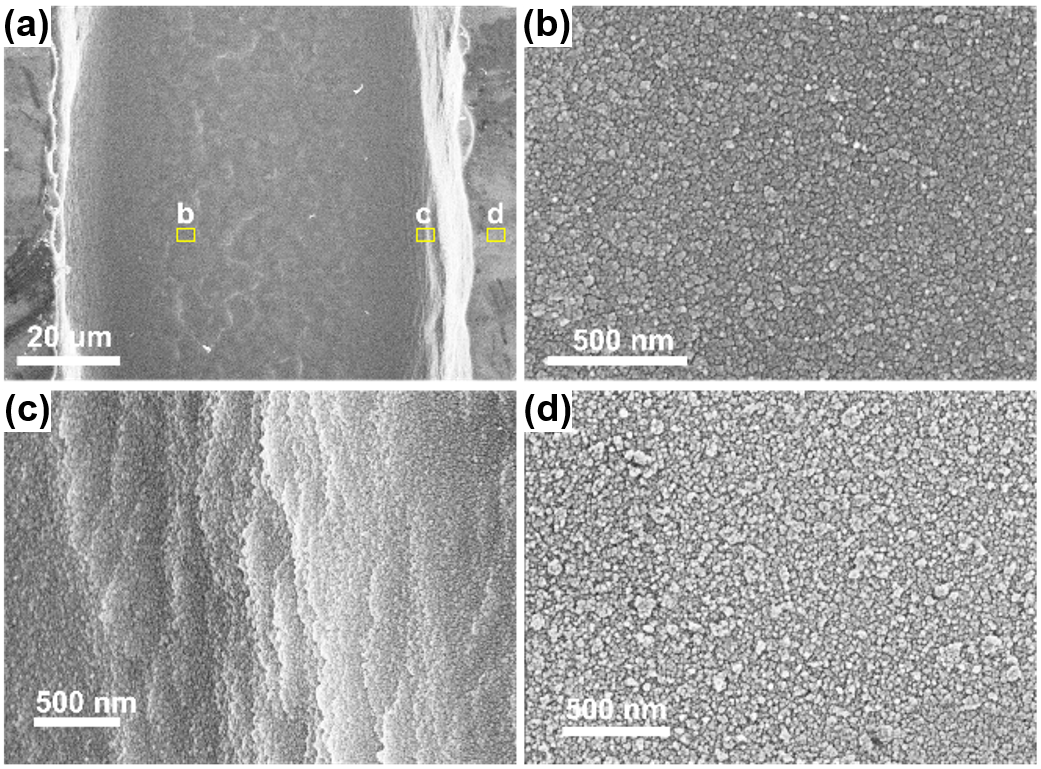


**Figure S8. Surface SEM images of the p-BN films (37.7 μm).**

Surface SEM images of the p-BN films deposited on the trenched Si substrate with the depth of 37.7 μm. b), c) and d) are marked area in a).

**
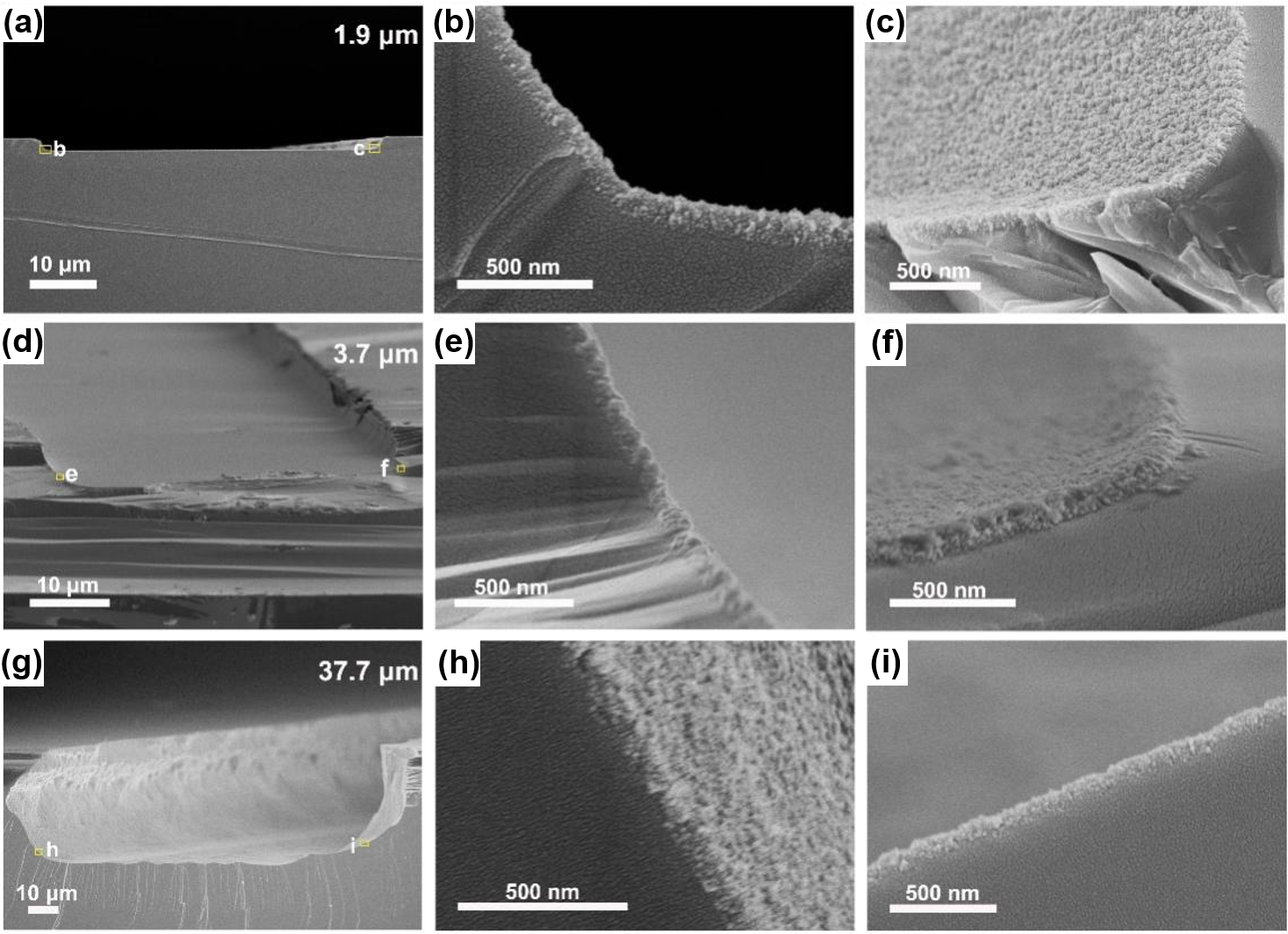
**

**Figure S9. Cross-sectional SEM images of the p-BN films.**

Images correspond to trench depths of a)-c) 1.9 μm, d)-f) 3.7 μm, and g)-i) 37.7 μm, respectively. b) and c) are magnified views of marked regions in a); e) and f) in d); h) and i) in g). The films exhibit uniform coverage even on high-aspect-ratio trench sidewalls with varying inclination angles, demonstrating excellent conformality for 3D structures.


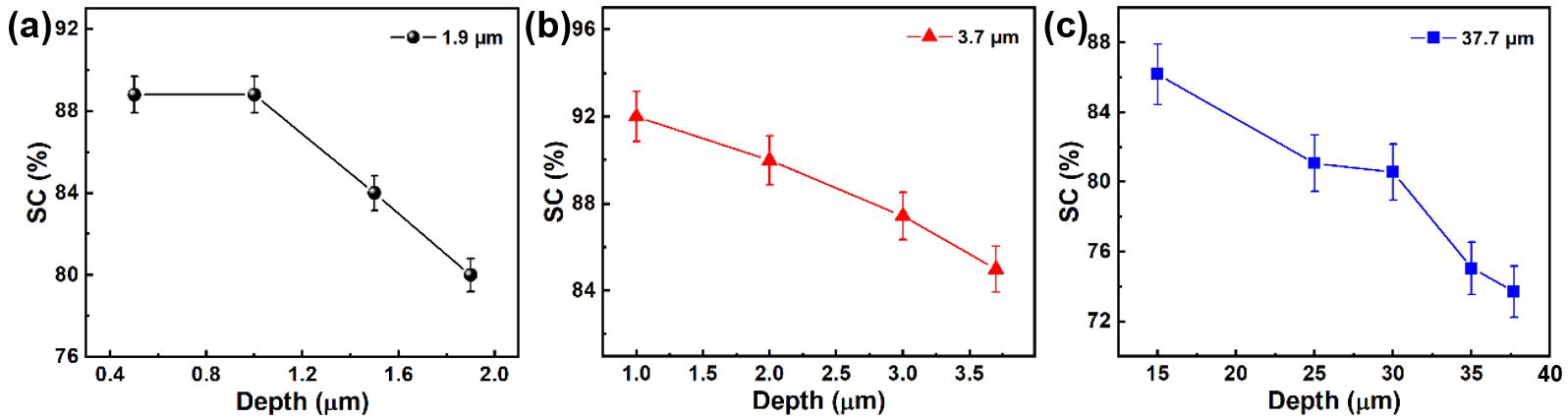


**Figure S10. Step coverage (SC) of p‑BN films as a function of trench depth.**

a) 1.9 μm, b) 3.7 μm, and c) 37.7 μm deep trenches. SC decreases moderately with increasing depth from 89% to 80% (1.9 μm), 92% to 85% (3.7 μm), and 86% to 74% (37.7 μm). The maintained coverage at lower sidewalls is attributed to the combined effects of RF plasma, elevated temperature, and applied pulsed bias during deposition. (20)


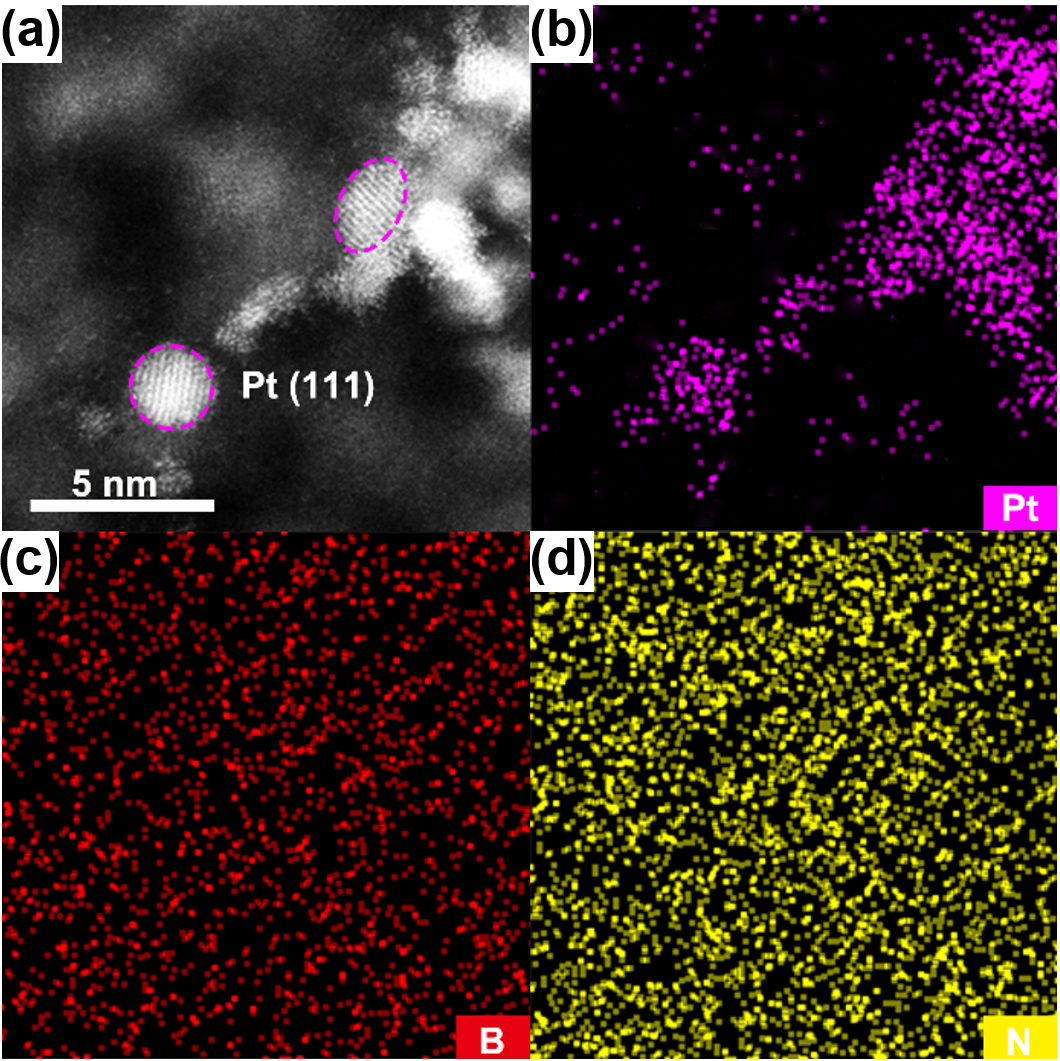


**Figure S11. STEM image and corresponding EDS elemental maps of the p‑BN film.**

a) STEM image and b)-d) EDS mapping of the p-BN film deposited at 100 V negative bias voltage.


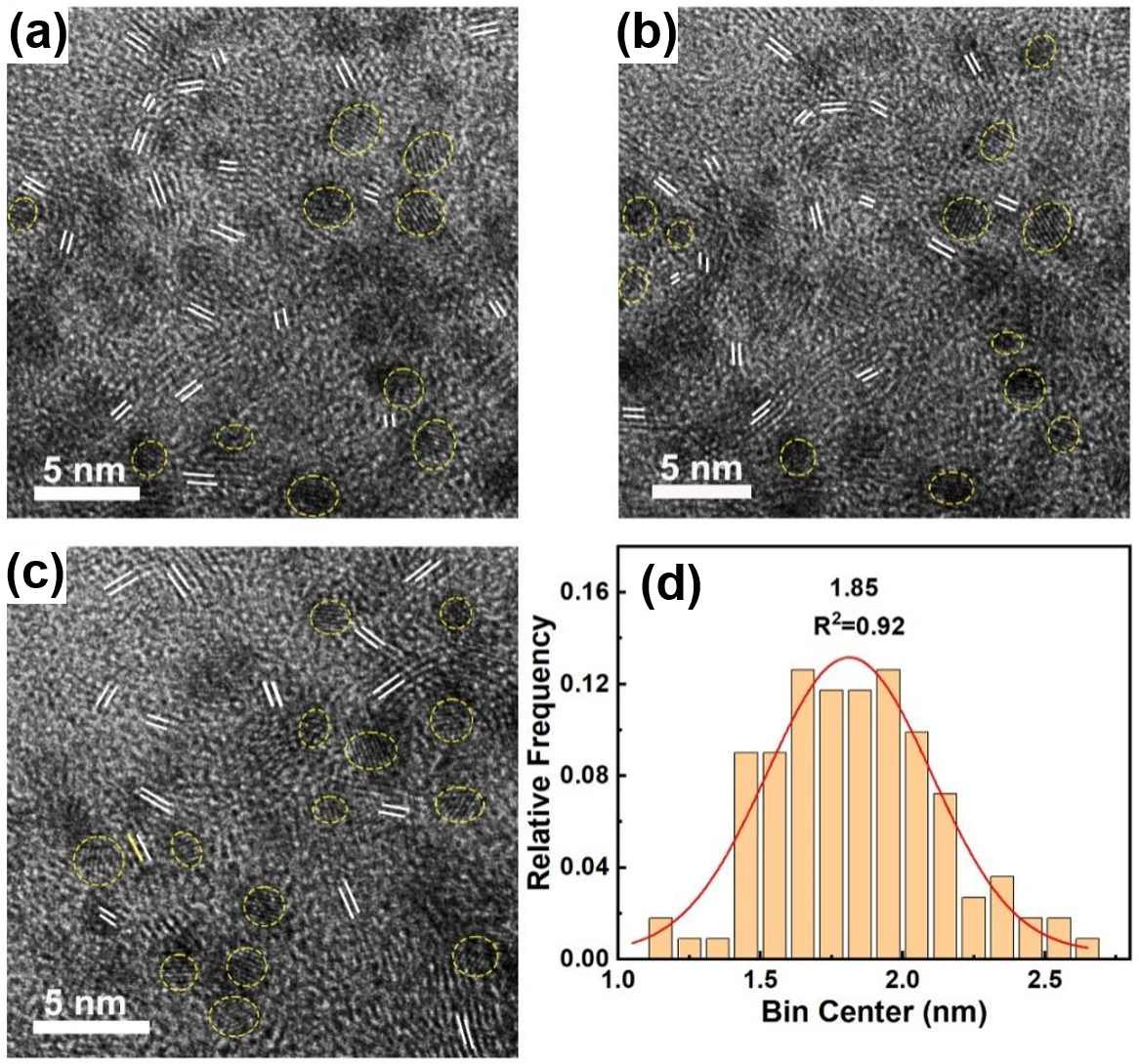


**Figure S12. HRTEM images of p-BN films.**

a)-c) HRTEM images and d) the statistically analysis of pore size distribution of p-BN films prepared at 100 V pulse negative bias voltage. (100)


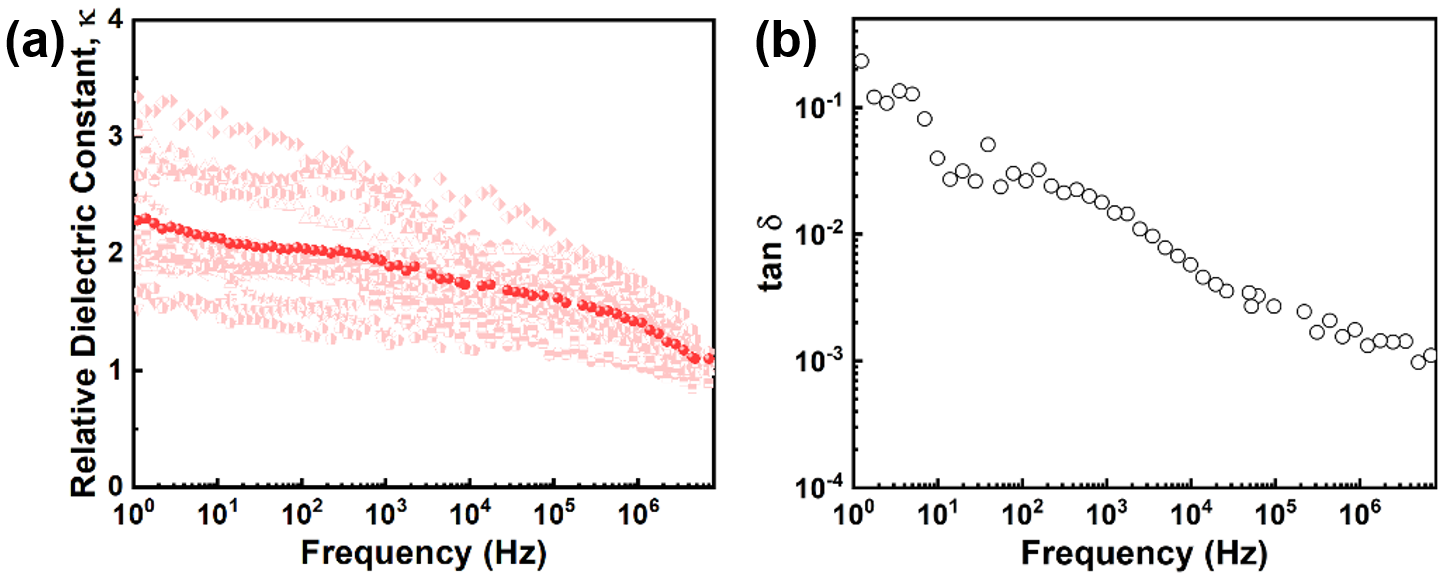


**Figure S13. Frequency dependent dielectric properties of p-BN films deposited at 100 V.**

a) Relative dielectric constant *κ* and b) dielectric loss factor tan δ measured over 1 Hz-7 MHz. The *κ* decreases with increasing frequency, reaching 1.63 at 100 kHz, while tan δ drops to ~0.001 at 7 MHz. (14)


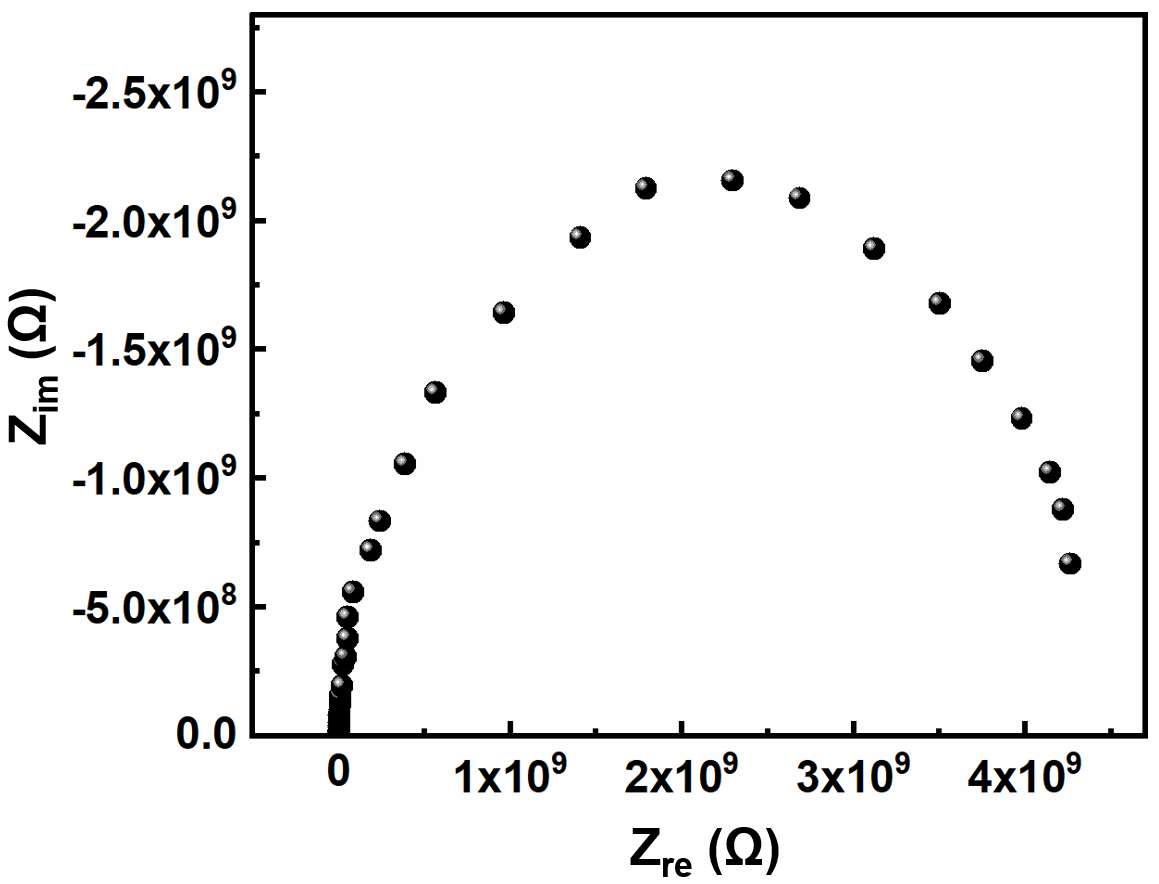


**Figure S14. Electrochemical impedance spectroscopy (EIS) of p‑BN films at room temperature.**

EIS (1 Hz-7 MHz) were acquired using a PARSTAT 3000A workstation under open‑circuit potential with a 15 mV (rms) AC signal. The single semicircle originates from bulk grains,^[1]^ with no discernible contribution from grain boundaries, indicating that grain boundary resistance is negligible compared to the high resistance of BN grains. Thus, the ultralow dielectric constant of p‑BN is attributed primarily to its pore structure rather than to grain boundary effects.


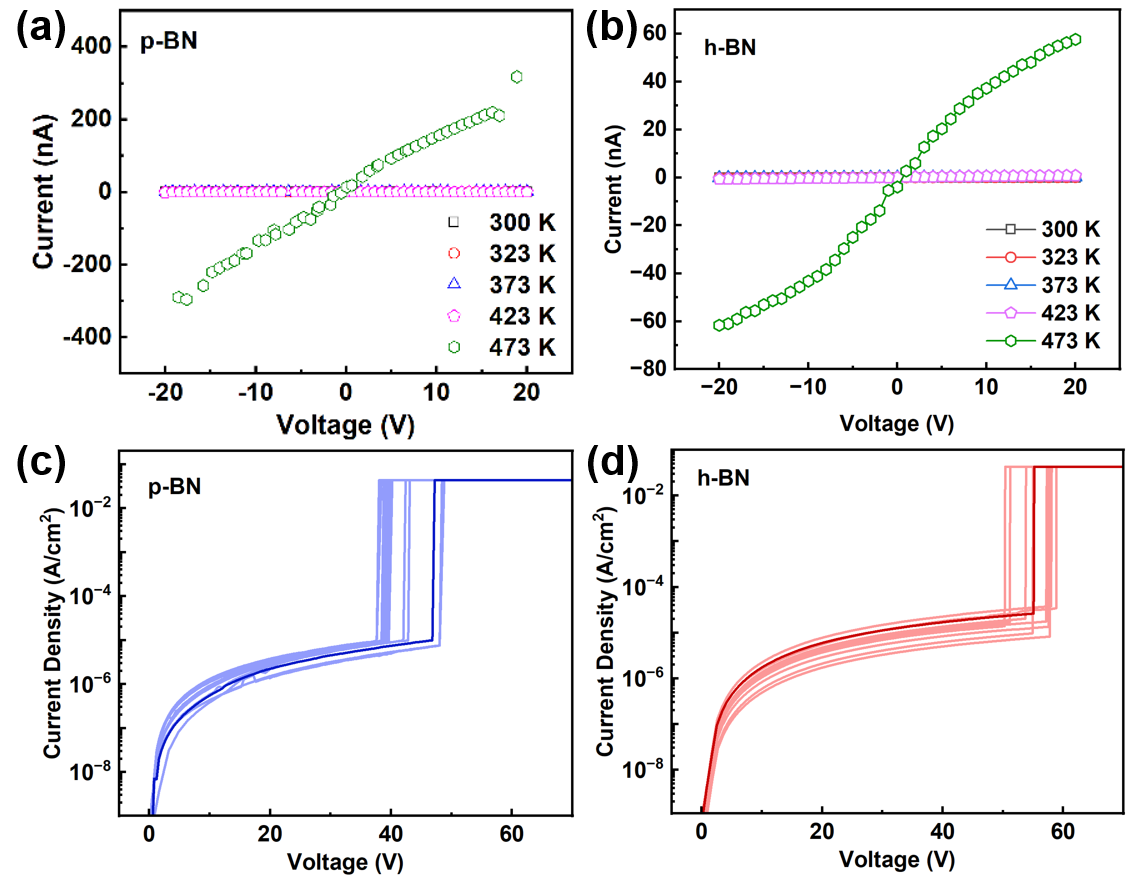


**Figure S15. Electrical characterization of p‑BN and dense h‑BN films.**

a) I‑V curves of p‑BN and b) h‑BN measured at different temperatures. c) and d) Current density-voltage (J‑V) characteristics for 100 nm p‑BN and 100 nm h‑BN, averaged over 14 and 9 devices, respectively. The breakdown field of p‑BN is 4.1 MV/cm, compared to 5.8 MV/cm for h‑BN. Corresponding leakage current densities are 1.17 µA/cm^2^ (p‑BN) and 1.15 µA/cm^2^ (h‑BN) at 10 V.


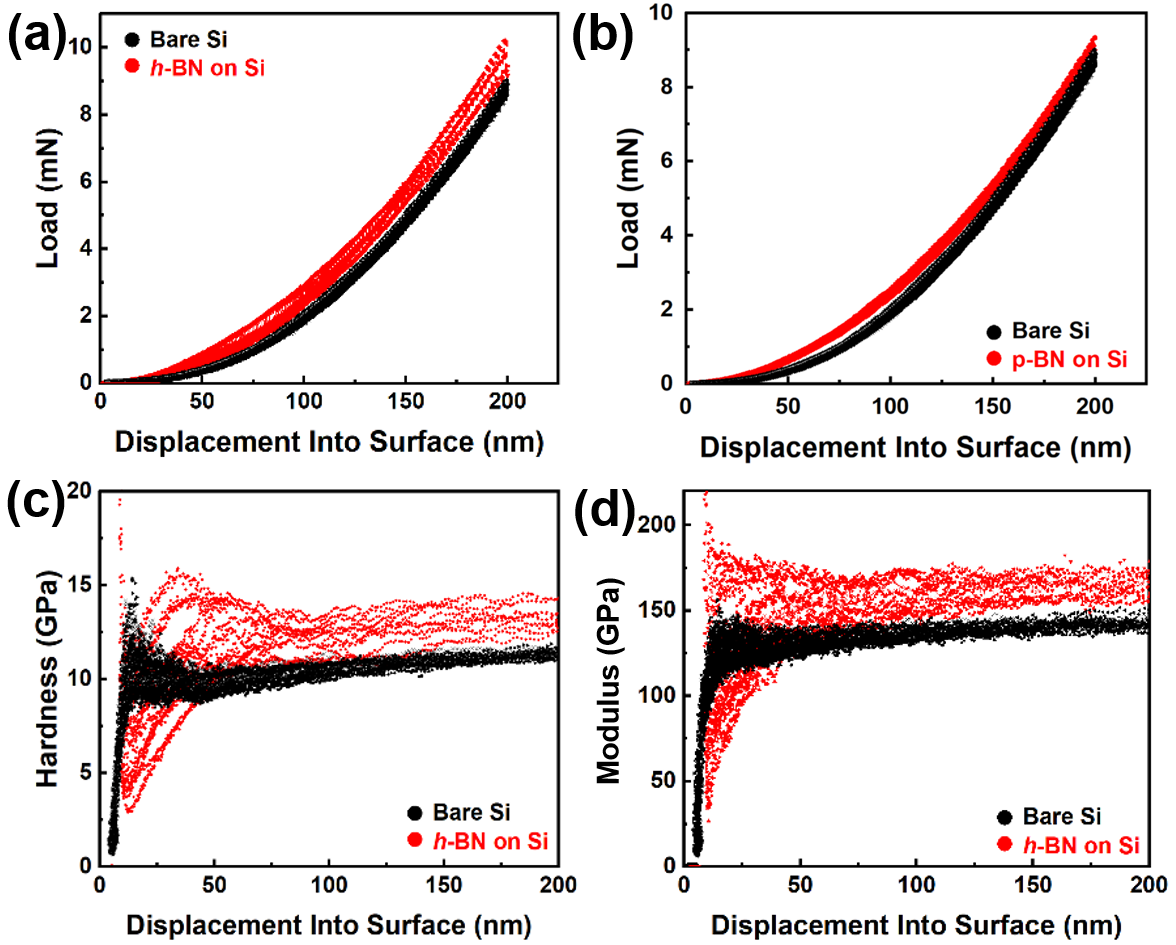


**Figure S16. Nanoindentation measurements of dense h-BN and p-BN films.**

a) and b) show the load-depth curves from nanoindentation measurements. Although the strength of p-BN is slightly lower than that of dense h-BN film, both are still higher than that of bare Si. c) and d) show the hardness and modulus curves from nanoindentation, respectively. The dense h-BN film on Si exhibits average hardness and modulus of 12.5 GPa and 171.5 GPa, compared to 10.5 GPa and 148.9 GPa for bare Si. (30)


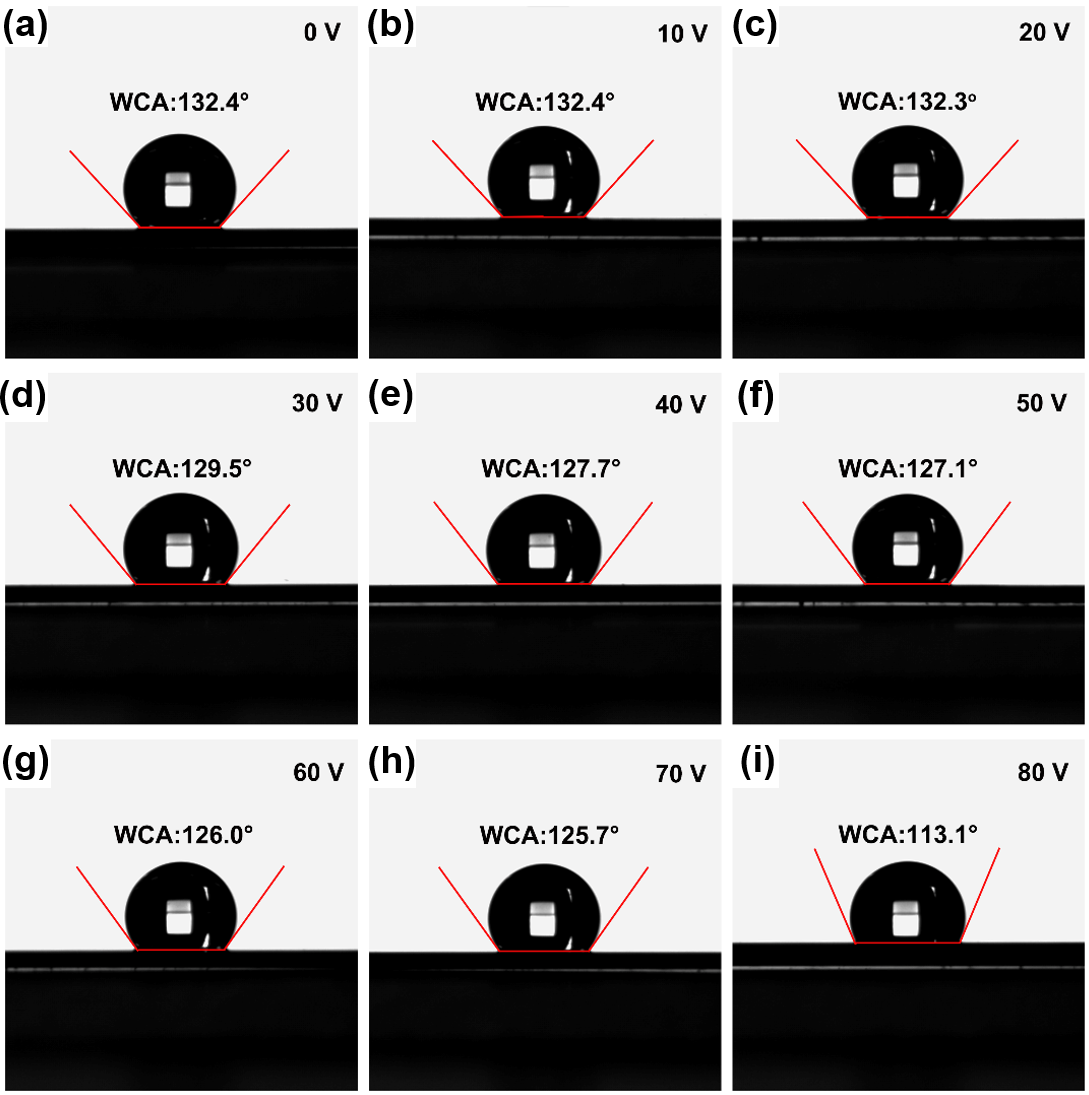


**Figure S17. Wettability of the p-BN films deposited with different bias voltages.**

a)-i) Images of the sessile water droplet on the surface of the p-BN film deposited with the pulsed negative bias voltage in the range of 0-80 V. The contact angle is decreased from 132.4° to 113.1° with the pulsed negative bias voltage increasing.


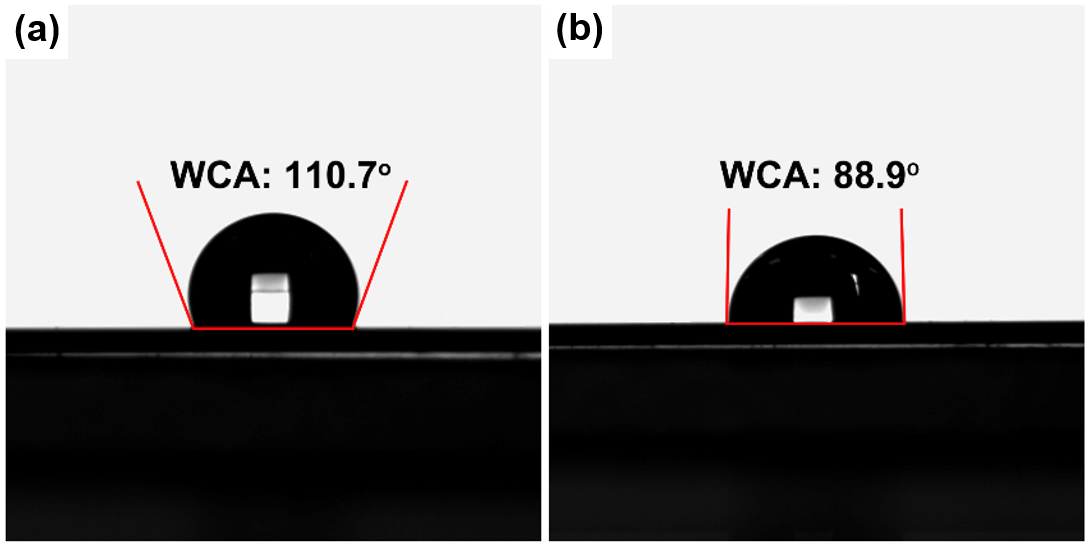


**Figure S18. Images of the sessile water droplet on the surface.**

Wettability of the a) p-BN film and b) dense BN film grown on Si substrate.


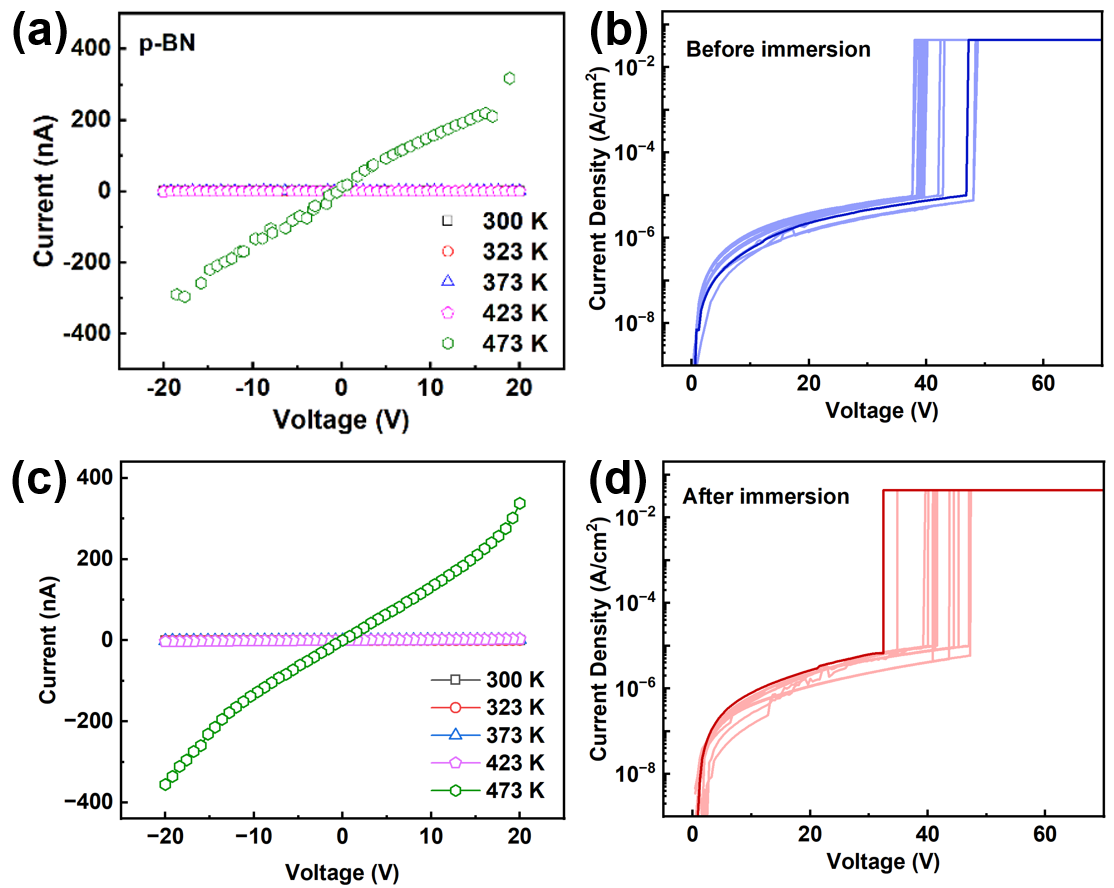


**Figure S19. Comparison of the electrical characteristics of the p-BN film before and after 48 h water immersion.**

a) I-V curve before immersion; b) breakdown curve before immersion; (12) c) I-V curve after immersion; d) breakdown curve after immersion. (12)


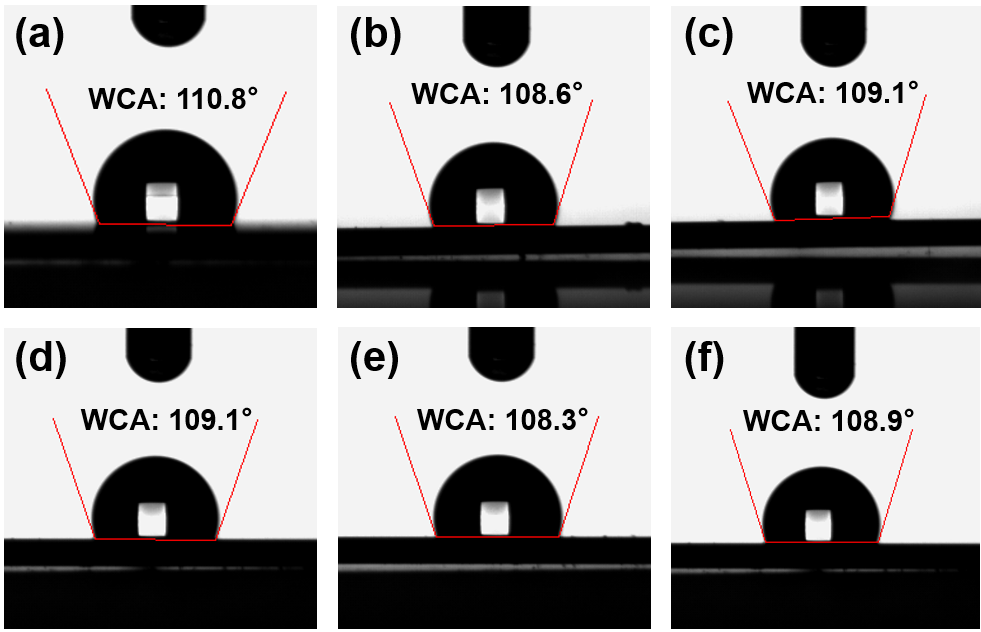


**Figure S20. Contact angle measurements (a)-(c) before and (d)-(f) after water immersion.**


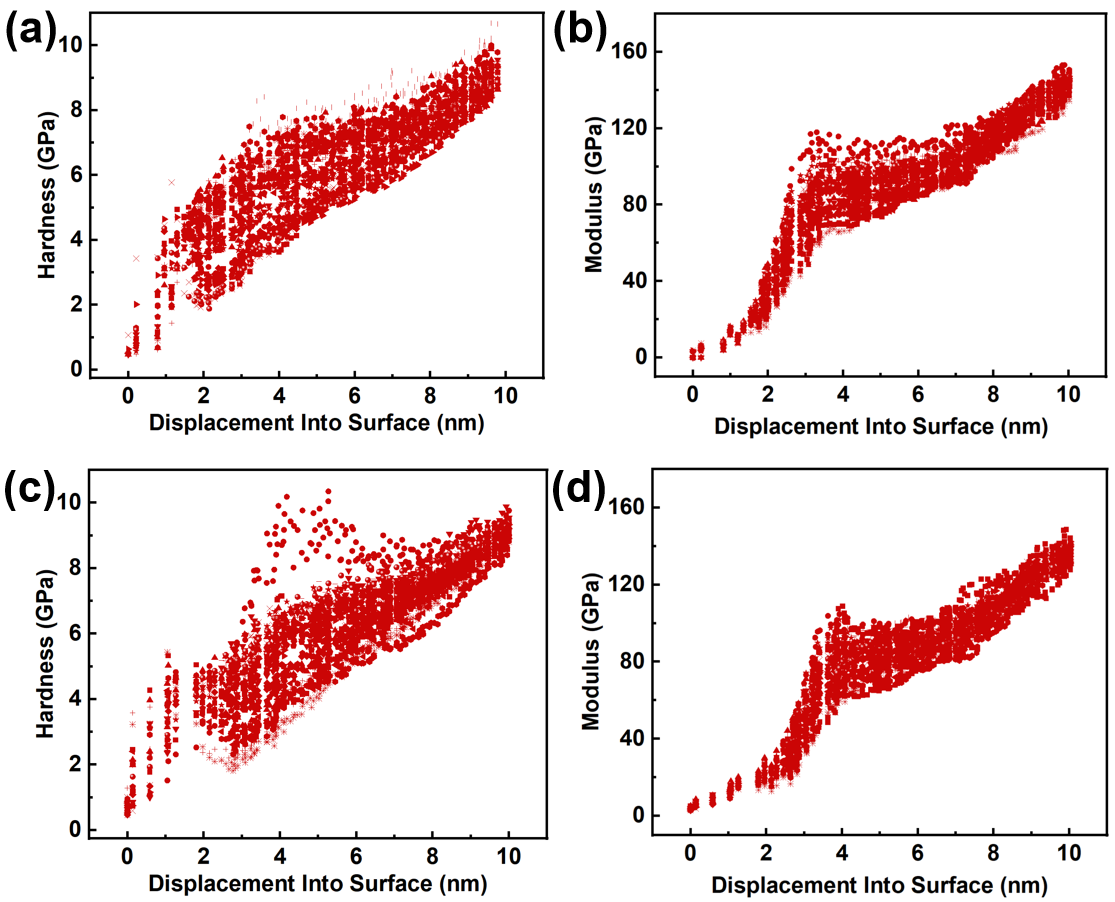


**Figure S21. Nanoindentation results of the p-BN film before and after 48 h water immersion.**

a) Hardness before immersion; b) Modulus before immersion; c) Hardness after immersion; d) Modulus after immersion. The average hardness values before and after immersion are 8.4 GPa and 8.3 GPa, respectively, and the average modulus values are 131.2 GPa and 128.5 GPa, respectively. (20)


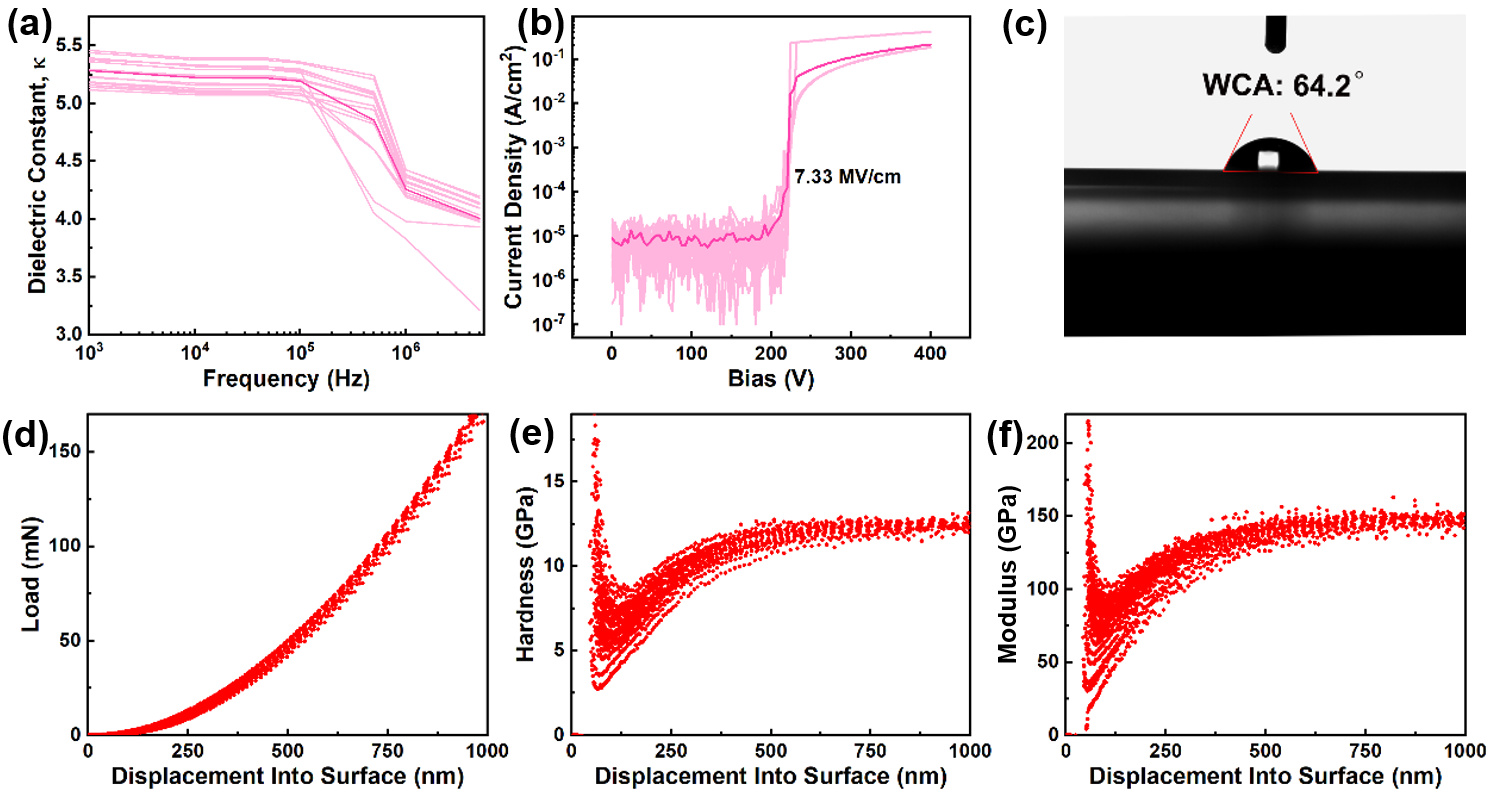


**Figure S22. Comparative electrical, wetting, and mechanical properties of a commercial SiO_2_ film (300 nm on Si).**

a) Relationship between relative dielectric constant and frequency (*κ* ≈ 5.19 at 100 kHz, higher than p‑BN and h‑BN). (14) b) J-V curve showing a breakdown field of 7.3 MV/cm and leakage current density of 9.2 µA/cm^2^ at 30 V. (14) c) Water contact angle of 64.2°, indicating lower hydrophobicity. d)-f) The nanoindentation measurements exhibits a hardness of 12.3 GPa and a modulus of 149.5 GPa. Overall, the SiO_2_ film exhibits weaker dielectric performance, reduced hydrophobicity, and lower mechanical stability compared to p‑BN and dense h‑BN films. (30)


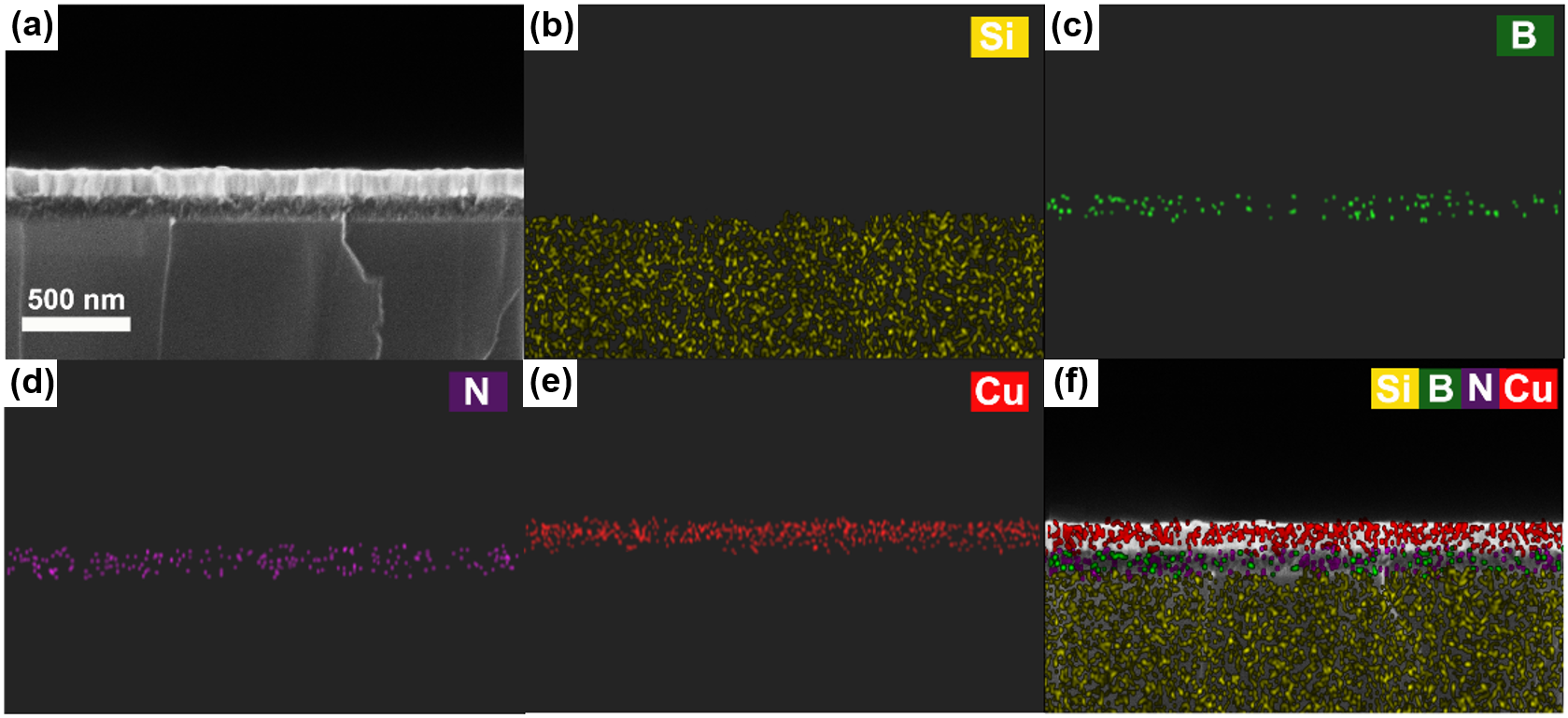


**Figure S23. Cross‑sectional SEM image and corresponding EDS elemental mapping of the Cu/p‑BN/Si stack.**

a) SEM image. b)-f) EDS maps showing the distribution of Si, B, N, and Cu. The elemental maps confirm that the deposited 100 nm Cu layer does not diffuse into the underlying p-BN film via the pore structure at room temperature.


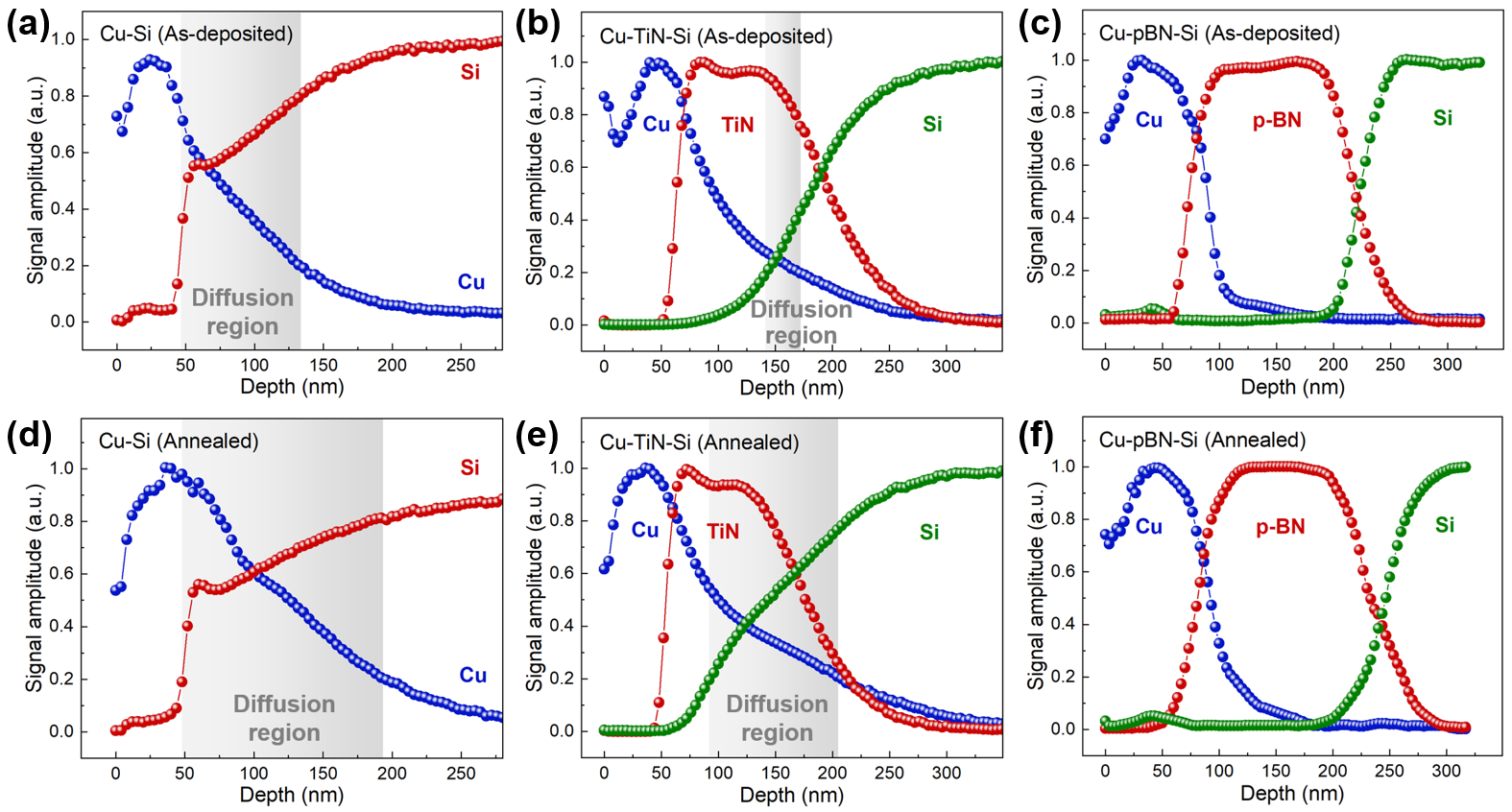


**Figure S24. Elemental depth profiles of Cu/Si, Cu/TiN/Si, and Cu/h-BN/Si stacks before and after annealing at 600 ℃.**

Depth profiling by secondary ion mass spectrometry (SIMS) was performed to evaluate different barrier materials. a)-c) show elemental depth profiles of the as‑deposited Cu/Si, Cu/TiN/Si, and Cu/h‑BN/Si stacks, respectively, while d)-f) present the corresponding profiles after vacuum annealing at 600 ℃ for 30 min. The gray shaded regions indicate Cu‑Si diffusion regions. The results demonstrate that p‑BN exhibits the best barrier performance. Although annealing aggravates Cu diffusion, p‑BN still effectively serves as a barrier layer.

**5. Tables**

**Table S1. The fitted results of the p-BN and dense h-BN films.**

|  | Thickness (nm) | Density (g/cm^3^) | RMS (nm) |
| --- | --- | --- | --- |
| p-BN | 96.30 | 1.41 | 1.85 |
| Dense h-BN | 56.93 | 2.28 | 0.66 |

**Table S2. Sidewall inclination angles and SC at different trench depths and positions.**

| Trench depth | 1.9 μm | | 3.7 μm | |
| --- | --- | --- | --- | --- |
| Depth position (μm) | 1.0 | ~1.9 | 1.0 | 2.0 |
| Sidewall inclination angle (°) | 111.2 | 124.7 | 108.5 | 135.0 |
| Step coverage | 89% | 80% | 92% | 90% |

**Table S3. Dielectric constant of the p-BN and dense h-BN films at different frequency.**

|  | 1 kHz | 5 kHz | 10 kHz | 50 kHz | 100 kHz | 1 MHz | 5 MHz |
| --- | --- | --- | --- | --- | --- | --- | --- |
| p-BN | 1.78 | 1.78 | 1.78 | 1.77 | 1.77 | 1.75 | 1.70 |
| Dense h-BN | 6.96 | 6.51 | 5.62 | 4.83 | 4.55 | 4.30 | 3.57 |

**Table S4. Comparison of various porous dielectric materials and continuous BN materials.**

|  | Dielectric Constant | Modulus  (GPa) | Hardness  (GPa) | Contact Angle  (°) |
| --- | --- | --- | --- | --- |
| Polyimide aerogel^[2]^ | 1.27-1.35 | 0.00781-  0.03353 |  | 83.7-107.6 |
| SiO_2_/PI nanofiber membranes  (porosity 90%)^[3]^ | 1.32-1.78 |  |  |  |
| PMSSQ  (porosity 25.1-61.2%)^[4]^ | 1.41-1.75 |  |  |  |
| Cross-linked SiO_2_ aerogel^[5]^ | 1.45-1.7 | 5.1 | 0.3 |  |
| Cyanoethyl cellulose^[6]^ | 1.56 |  |  |  |
| PEN/SiO_2_ foams^[7]^ | 1.71 | 1.96±0.07 |  | 98.1 |
| Electrospun epoxy film^[8]^ | 1.90 | 0.50±0.08 | 0.17±0.03 | 145 |
| FG/FPBO^[9]^ | 2.02 | 1.98 |  | 83.7±1 |
| pSiCOH  (porosity 29%)^[10]^ | 2.05 | 3.3 | 0.28 |  |
| pSiCOH^[11]^ | 2.02 _@100 kHz_ | 5.3 |  |  |
| t-FG/f-PI^[12]^ | 2.09 | 4.79 |  |  |
| SiLK^[13]^ | 2.20 |  |  |  |
| HSQ  (porosity 46%)^[14]^ | 2.20 |  |  |  |
| Sandwich-type polyimide film  (porosity 22.2-66.7%)^[15]^ | 2.24-2.81 | 1.2-1.4 |  | 99.8-121.7 |
| Fluorinated polyimide/POSS^[16]^ | 2.58 | 1.8 |  |  |
| a-BN film^[17]^ | 5.9 at 1kHz |  |  |  |
| a-BN film^[18]^ | 1.78 at 100kHz  1.18 at 1MHz |  | 11.31 |  |
| h-BN film^[19,20]^ | 2-4 | 120-180 | 10-14 |  |
| h-BN film^[18]^ | 3.28 at 100kHz  2.87 at 1MHz |  |  |  |
| h*-*BN film  (This work) | 4.36 at 100 kHz  4.01 at 1MHz | 171.5 | 12.5 | 88.9 |
| Porous BN film  (This work) | 1.77 at 100 kHz  1.75 at 1MHz | 161.4 | 11.7 | 110.7 |

**Table S5. Comparison of dielectric constants of the p-BN film before and after water immersion.**

|  | 1 kHz | 5 kHz | 10 kHz | 50 kHz | 100 kHz | 1 MHz | 5 MHz |
| --- | --- | --- | --- | --- | --- | --- | --- |
| p-BN  (before) | 1.78 | 1.78 | 1.78 | 1.77 | 1.77 | 1.75 | 1.70 |
| p-BN  (after) | 1.79 | 1.79 | 1.78 | 1.76 | 1.76 | 1.75 | 1.68 |

**References**

[1] H. L. Tuller, S. J. Litzelman, and W. Jung, “Micro-ionics: next generation power sources,” *Physical Chemistry Chemical Physics* 11, no. 17 (2009): 3023, https://doi.org/10.1039/B901906E

[2] T. Wu, J. Dong, F. Gan, Y. Fang, X. Zhao, and Q. Zhang, “Low dielectric constant and moisture-resistant polyimide aerogels containing trifluoromethyl pendent groups,” *Applied Surface Science* 440, (2018): 595, https://doi.org/10.1016/j.apsusc.2018.01.132

[3] L. Liu, F. Lv, P. Li, et al., “Preparation of ultra-low dielectric constant silica/polyimide nanofiber membranes by electrospinning,” *Composites Part A: Applied Science and Manufacturing* 84, (2016): 292, https://doi.org/10.1016/j.compositesa.2016.02.002

[4] C. Wang, T. M. Wang, and Q. H. Wang, “Ultralow-dielectric, nanoporous poly (methyl silsesquioxanes) films templated by a self-assembled block copolymer upon solvent annealing,” *Journal of Polymer Research* 26, no. 1 (2018): 5, https://doi.org/10.1007/s10965-018-1650-z

[5] H. Choi, T. Kim, T. Kim, et al., “Ultralow dielectric cross-linked silica aerogel nanocomposite films for interconnect technology,” *Applied Materials Today* 28, (2022): 101536, https://doi.org/10.1016/j.apmt.2022.101536

[6] B. Wang, H. Kang, H. Yang, J. Xie, and R. Liu, “Preparation and dielectric properties of porous cyanoethyl cellulose membranes,” *Cellulose* 26, no. 2 (2019): 1261, https://doi.org/10.1007/s10570-018-2132-5

[7] Q. Qi, P. Zheng, Y. Lei, and X. Liu, “Design of bi-modal pore structure polyarylene ether nitrile/SiO_2_ foams with ultralow-k dielectric and wave transparent properties by supercritical carbon dioxide,” *Composites Part B: Engineering* 173, (2019): 106915, https://doi.org/10.1016/j.compositesb.2019.106915

[8] Y. Zhang, C. Zhao, J. Liu, and H. Na, “Preparation and characterization of ultralow dielectric and fibrous epoxy thermoset cured with poly (arylene ether ketone) containing phenolic hydroxyl groups,” *European Polymer Journal* 109, (2018): 110, https://doi.org/10.1016/j.eurpolymj.2018.08.059

[9] Z. Yu, S. Wu, C. Li, et al., “Ultra-low dielectric constant fluorinated graphene/polybenzoxazole composite films with excellent thermal stabilities and mechanical properties,” *Composites Part A: Applied Science and Manufacturing* 145, (2021): 106387, https://doi.org/10.1016/j.compositesa.2021.106387

[10] A. Grill, “Plasma enhanced chemical vapor deposited SiCOH dielectrics: from low-k to extreme low-k interconnect materials,” *Journal of Applied Physics* 93, no. 3 (2003): 1785, https://doi.org/10.1063/1.1534628

[11] P. Verdonck, C. Wang, Q. T. Le, et al., “Advanced PECVD SiCOH low-k films with low dielectric constant and/or high Young’s modulus,” *MAM2013, March 10-13, Leuven, Belgium* 120, (2014): 225, https://doi.org/10.1016/j.mee.2013.10.028

[12] X. Yin, Y. Feng, Q. Zhao, et al., “Highly transparent, strong, and flexible fluorographene/fluorinated polyimide nanocomposite films with low dielectric constant,” *Journal of Materials Chemistry C* 6, no. 24 (2018): 6378, https://doi.org/10.1039/C8TC00998H

[13] Y. N. Su, J. H. Shieh, B. C. Perng, S. M. Jang, and M. S. Liang, in *Proceedings of the IEEE 2005 International Interconnect Technology Conference, 2005.* (2005): 54-56

[14] H.-J. Lee, C. L. Soles, D.-W. Liu, et al., “Structural characterization of porous low-k thin films prepared by different techniques using X-ray porosimetry,” *Journal of Applied Physics* 95, no. 5 (2004): 2355, https://doi.org/10.1063/1.1641955

[15] Y. Ma, L. Xu, Z. He, et al., “Tunable dielectric and other properties in high-performance sandwich-type polyimide films achieved by adjusting the porous structure,” *Journal of Materials Chemistry C* 7, no. 24 (2019): 7360, https://doi.org/10.1039/C9TC02017A

[16] C. Wang, W. Chen, C. Xu, X. Zhao, and J. Li, “Fluorinated polyimide/POSS hybrid polymers with high solubility and low dielectric constant,” *Chinese Journal of Polymer Science* 34, no. 11 (2016): 1363, https://doi.org/10.1007/s10118-016-1845-x

[17] N. R. Glavin, C. Muratore, M. L. Jespersen, et al., “Amorphous Boron Nitride: A Universal, Ultrathin Dielectric For 2D Nanoelectronics,” *Advanced Functional Materials* 26, no. 16 (2016): 2640, https://doi.org/10.1002/adfm.201505455

[18] S. Hong, C.-S. Lee, M.-H. Lee, et al., “Ultralow-dielectric-constant amorphous boron nitride,” *Nature* 582, no. 7813 (2020): 511, https://doi.org/10.1038/s41586-020-2375-9

[19] V. A. S. Kandadai, V. Gadhamshetty, and B. K. Jasthi, “Effect of buffer layer and substrate growth temperature on the microstructural evolution of hexagonal boron nitride thin films,” *Surface and Coatings Technology* 447, (2022): 128805, https://doi.org/10.1016/j.surfcoat.2022.128805

[20] S. K. Jang, J. Youn, Y. J. Song, and S. Lee, “Synthesis and Characterization of Hexagonal Boron Nitride as a Gate Dielectric,” *Scientific Reports* 6, no. 1 (2016): 30449, https://doi.org/10.1038/srep30449
